# DNN Surrogate-Assisted Multi-Objective Optimization for Inverse Process Design of SBS-Modified Asphalt Gels

**DOI:** 10.3390/gels12100901

**Published:** 2026-10-07

**Authors:** Zongyuan Wu, Xiaoyu Ma, Jiaming Yang, Mengxin Qiu, Yang Sun, Decai Wang

**Affiliations:** 1 School of Civil Engineering and Communication, North China University of Water Resources and Electric Power, Zhengzhou 450045, China; 2 Provincial Engineering Research Center for New Energy Vehicle Intelligent Control and Simulation Test Technology of Sichuan, Chengdu 610039, China

**Keywords:** SBS-modified asphalt, polymer gel, surrogate-assisted optimization, multi-objective optimization, processing parameter optimization

## Abstract

Styrene–butadiene–styrene (SBS)-modified asphalt is a typical physical polymer gel whose macroscopic performance is governed by the coupled effects of processing parameters, material composition, and microstructural characteristics. While machine learning has enabled accurate forward prediction of gel properties, a systematic method to inversely determine optimal processing conditions from desired performance targets remains lacking. This study proposes a surrogate-assisted multi-objective optimization framework that bridges forward prediction and inverse design for SBS-modified asphalt gels. A single multi-output deep neural network (DNN) surrogate was trained to predict penetration, softening point, ductility, and viscosity at 135 °C. After correcting the data-splitting procedure, the independent test-set R^2^ values ranged from approximately 0.981 to 0.994 across the four outputs. NSGA-II was then used to identify scenario-specific Pareto-optimal processing conditions. Three representative Pareto solutions, including the balanced, high-softening-point, and high-ductility solutions, were experimentally validated. Across the 12 prediction–measurement comparisons, the relative deviations ranged from approximately 0.86% to 2.62%. The framework provides a bounded-domain approach for converting forward property prediction into experimentally testable processing recommendations for SBS-modified asphalt gels.

## 1. Introduction

Paving-grade asphalt modified with styrene–butadiene–styrene (SBS) behaves as a physical gel rather than as a simple polymer dispersion [1]. During high-temperature mixing, the polybutadiene mid-blocks of SBS become compatible with the light fractions of the binder, while the polystyrene end-blocks aggregate into glassy nanodomains upon cooling [2,3]. These domains act as reversible physical crosslinks, and the flexible polybutadiene segments bridge them into a three-dimensional network [4]. This network provides elastic recovery under repeated traffic loading and temporary flow during paving, because the physical crosslinks can dissociate and re-form as temperature changes [5]. The fatigue resistance and service performance of SBS-modified asphalt mixtures are therefore closely tied to the integrity of this gel network [6].

Polymer modifiers used in asphalt can be broadly grouped into elastomers and plastomers. Elastomeric modifiers, including SBS and styrene–butadiene rubber, improve elastic recovery and are generally beneficial for both high-temperature rutting resistance and low-temperature cracking resistance [2]. Plastomeric modifiers, such as polyethylene and polypropylene, increase stiffness and high-temperature performance but often reduce flexibility. SBS belongs to the thermoplastic elastomer family, specifically the styrenic block copolymers. Its molecular architecture combines rigid polystyrene end-blocks and a rubbery polybutadiene middle block, which is the reason SBS can form a reversible physical network rather than a permanently crosslinked system [7].

The performance of the resulting gel is controlled by a coupled set of process and material variables [8]. Shear temperature affects binder viscosity and the mobility of polymer chains, and thus the kinetics of SBS swelling and dispersion [9]. Shear rate determines the mechanical energy available to break down SBS agglomerates and to distribute the polymer phase [10]. Shear time controls how far swelling and homogenization proceed before the binder is cooled [11]. The base asphalt chemistry governs compatibility between the polymer and the maltene/asphaltene fractions [12], while the SBS molecular architecture and dosage determine the density and continuity of the physical network [13,14]. Post-preparation descriptors such as the average SBS domain size and FTIR-derived polystyrene index ($I_{PS}$), polybutadiene index ($I_{PB}$), and butadiene index ($I_{B}$) provide additional information on dispersion quality and polymer–asphalt interactions [15,16,17].

Because these variables interact nonlinearly, one-factor-at-a-time experiments and empirical regression models cannot map the full process–property space [18]. Conventional mix design relies on trial-and-error, consumes time and material, and explores only a small part of the feasible processing window; interaction effects among temperature, shear rate, shear time, and dosage are easily missed [19].

The Materials Genome Initiative has encouraged a shift from trial-and-error toward data-driven design in materials science [20,21]. In asphalt research, this shift is visible in the construction of multi-scale databases and computational models [22,23,24,25]. Machine learning has become a practical tool for learning high-dimensional nonlinear relationships in these databases [26,27]. Neural networks have been used to estimate penetration, softening point, ductility, and viscosity of modified binders [28]. Classical models with genetic hyperparameter optimization have been applied to cold recycled mixtures [29], and specialized models have been developed for rheological properties of polymer-modified binders [30,31]. Comparative studies have examined single and ensemble learners for complex shear modulus and phase angle [32], while deep learning has been used to characterize SBS-modified asphalt microstructures [33]. Artificial neural networks have also predicted frequency- and temperature-dependent dynamic modulus and phase angle [34]. These studies confirm that forward property prediction is now well established.

What remains less developed is the inverse problem: given desired performance targets, which processing conditions should be used? Most existing studies predict properties from known inputs, whereas industrial formulation requires the opposite direction [35]. The problem is inherently multi-objective because asphalt properties conflict. Increasing SBS network density can raise softening point but also viscosity, and may reduce ductility. A single weighted objective would impose an arbitrary preference before the trade-offs are known. Multi-objective evolutionary optimization is therefore a natural choice.

NSGA-II is well suited to this task because it combines fast non-dominated sorting, crowding-distance maintenance, and elitist selection [36]. Surrogate-assisted NSGA-II has already been used to accelerate Superpave mix design [37], to balance mechanical, economic, and environmental objectives in hydraulic asphalt concrete [38], and to consider performance, environmental impact, and life-cycle cost in asphalt mix design [39]. Other evolutionary algorithms have also been coupled with machine learning for mix design automation [40]. Tutorials and monographs provide the theoretical basis for Pareto-based decision making when no unique scalar objective exists [41,42]. In the present work, a single multi-output DNN is trained on 864 experimental records and then frozen as the performance evaluator inside NSGA-II. The search is performed separately for eight material scenarios formed by two base asphalts and four SBS types, so that categorical materials are not mixed artificially. The decision variables are limited to settings that can be specified before preparation: the SBS dosage ranges from 3.0% to 7.5%, the shear temperature ranges from 170 °C to 190 °C, the shear rate ranges from 2000 rpm to 6000 rpm, and the shear time ranges from 30 min to 120 min. Particle size and FTIR indices are retained only as descriptive variables and are not optimized, because they cannot be set independently in production.

Three contributions distinguish this study from earlier forward-prediction work. First, it integrates a DNN surrogate with NSGA-II for inverse process design of SBS-modified asphalt gels. Second, it analyzes the Pareto trade-offs among softening point, ductility, and viscosity and applies an engineering screen to identify practically relevant candidates. Third, it validates a balanced Pareto solution experimentally, showing that the recommended conditions are physically achievable. The framework is demonstrated for two base asphalts and four SBS types; its conclusions are therefore bounded by this material and process domain and should not be generalized to other systems without retraining and validation.

## 2. Results and Discussion

### 2.1. Surrogate Model Validation

The 864 complete records were divided into training, validation, and test subsets using the fixed random seed described in Section 2.3.1. The 70%/15%/15% split produced 604 training records, 130 validation records, and 130 test records. The validation subset was used for early stopping, while the test subset was held out from model selection and used only for final performance reporting. This separation ensures that the reported accuracy reflects generalization within the bounded experimental domain rather than fit to the training data.

The predictive performance of the single multi-output DNN on the independent test set is summarized in Table 1. The test-set R^2^ values ranged from 0.9807 to 0.9939 across the four target properties, indicating consistently high predictive accuracy within the investigated material and processing domain. In addition to RMSE and MAE, the 95th percentile of the absolute prediction error, P_95_(|e|), was calculated to characterize the upper tail of the residual distribution. The P95 values were 0.382 (0.1 mm) for penetration, 1.187 °C for softening point, 0.533 cm for ductility, and 0.071 Pa·s for viscosity at 135 °C. Thus, 95% of the absolute prediction errors for each target remained below the corresponding P95 threshold on the independent test set. These results support the use of the trained DNN as a surrogate model for the subsequent multi-objective optimization within the investigated design domain.

The important methodological point is the use of one shared multi-output network rather than four independent single-output models. A single predictor simplifies deployment because every NSGA-II evaluation uses the same preprocessing pipeline, network state, and inference call. It also prevents inconsistencies that would arise from different model families, each with its own bias and variance characteristics, when the optimizer moves across the objective landscape. The high accuracy of all four outputs means that the optimization stage can focus on objective interactions instead of compensating for one clearly weaker predictor.

**Figure 1:**
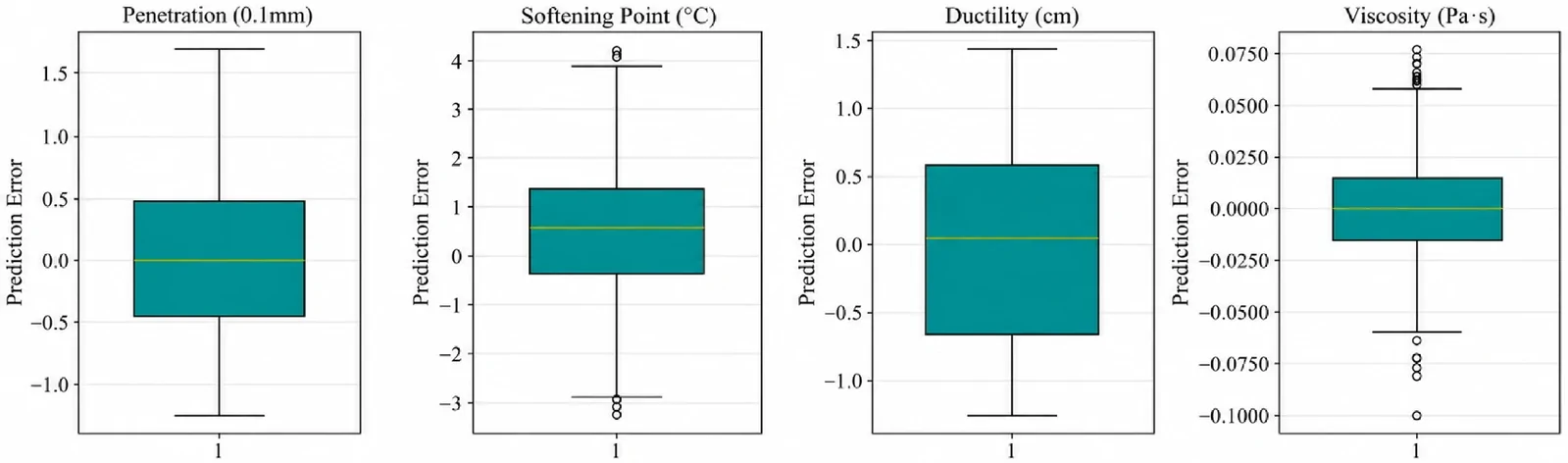
Boxplots of prediction errors for the deep neural network surrogate on the validation set. Each boxplot shows the median, interquartile range, and 1.5 times the interquartile range.

The prediction errors on the validation set are presented in Figure 1. All targets exhibited small and symmetrically distributed errors, with median errors close to zero and narrow interquartile ranges. The penetration predictions showed the smallest error spread, with a median absolute error of 0.28 (0.1 mm). The softening point predictions had a median absolute error of 0.49 °C. The ductility predictions had a median absolute error of 0.41 cm. The viscosity predictions had a median absolute error of 0.05 Pa·s. The maximum absolute errors were less than 1.5 times the median for all targets, indicating that the errors were well-behaved and did not contain extreme outliers.

**Figure 2:**
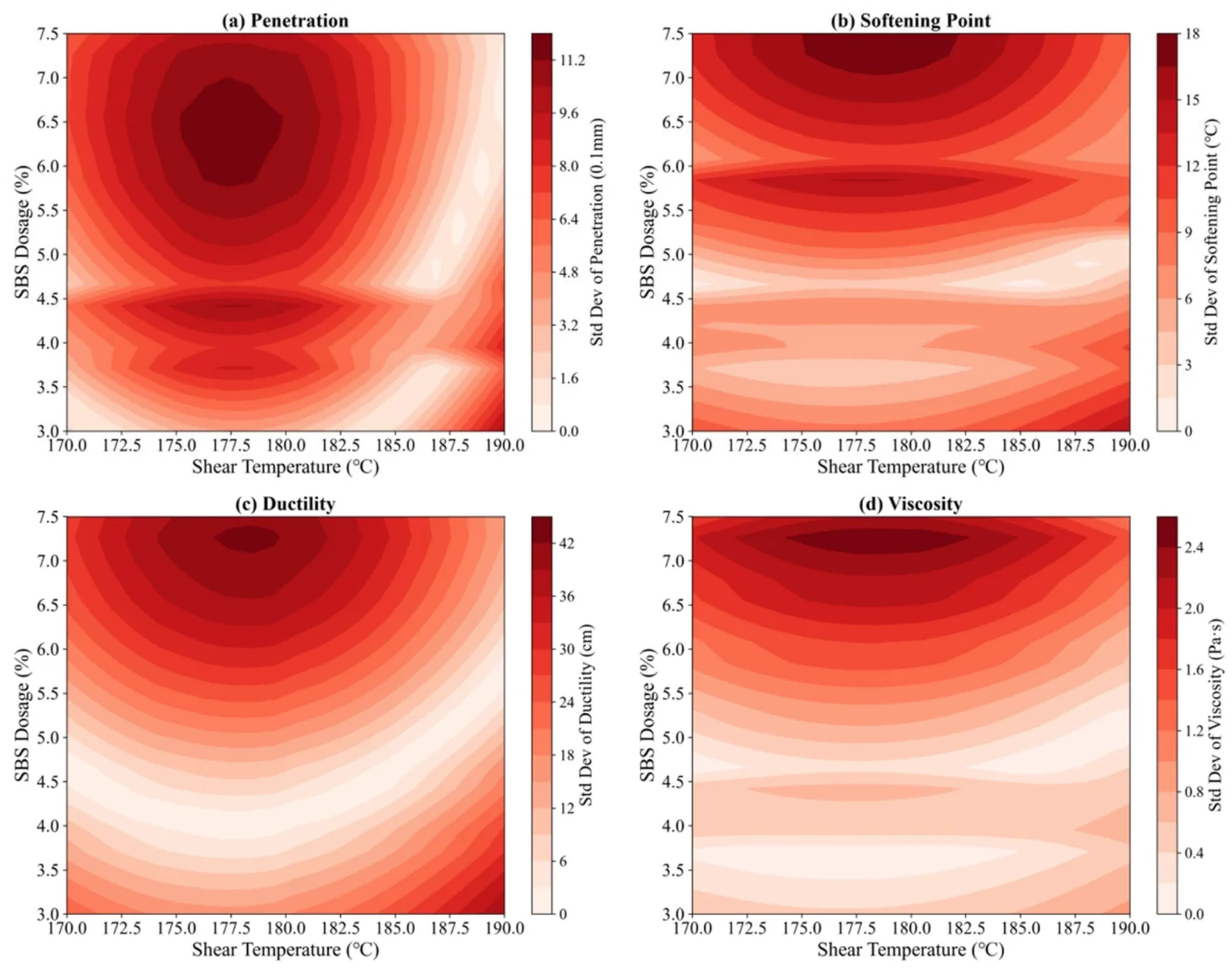
Prediction variance heatmap of softening point over shear temperature and SBS dosage. Contours represent the standard deviation of predictions from ten independently trained models. Lighter colors indicate lower variance and higher prediction confidence.

To further assess prediction stability, the prediction variance of softening point across the temperature-dosage space is shown in Figure 2. The standard deviation was calculated from ten independently trained DNNs with different random seeds. The standard deviation was generally below 0.5 °C in the central region of the design space, where the training data were concentrated. Slightly higher values were observed at extreme combinations, such as low temperature with high dosage or high temperature with low dosage. These regions correspond to areas of lower data density, where the model showed greater uncertainty. The low variance across the central design space confirmed that the surrogate predictions were stable and reliable for optimization use.

The accuracy should nevertheless be interpreted in the context of the dataset. The records form a dense experimental grid with repeated material combinations, and the test points remain inside the same bounded design domain. The reported R^2^ and RMSE values therefore demonstrate interpolation accuracy within the covered process and composition space rather than universal generalization to entirely new binder chemistries or process regimes. This limitation is not a reason to reject the surrogate. It defines the scope within which the optimizer should be trusted. Within this bounded domain, the combination of high R^2^ values, low and symmetric error distributions, and low prediction variance across multiple initializations confirms that the DNN surrogate is sufficiently accurate and stable to serve as the performance evaluator inside the NSGA-II optimization loop.

### 2.2. Optimization Convergence and Pareto Front Analysis

#### 2.2.1. Optimization Convergence

**Figure 3:**
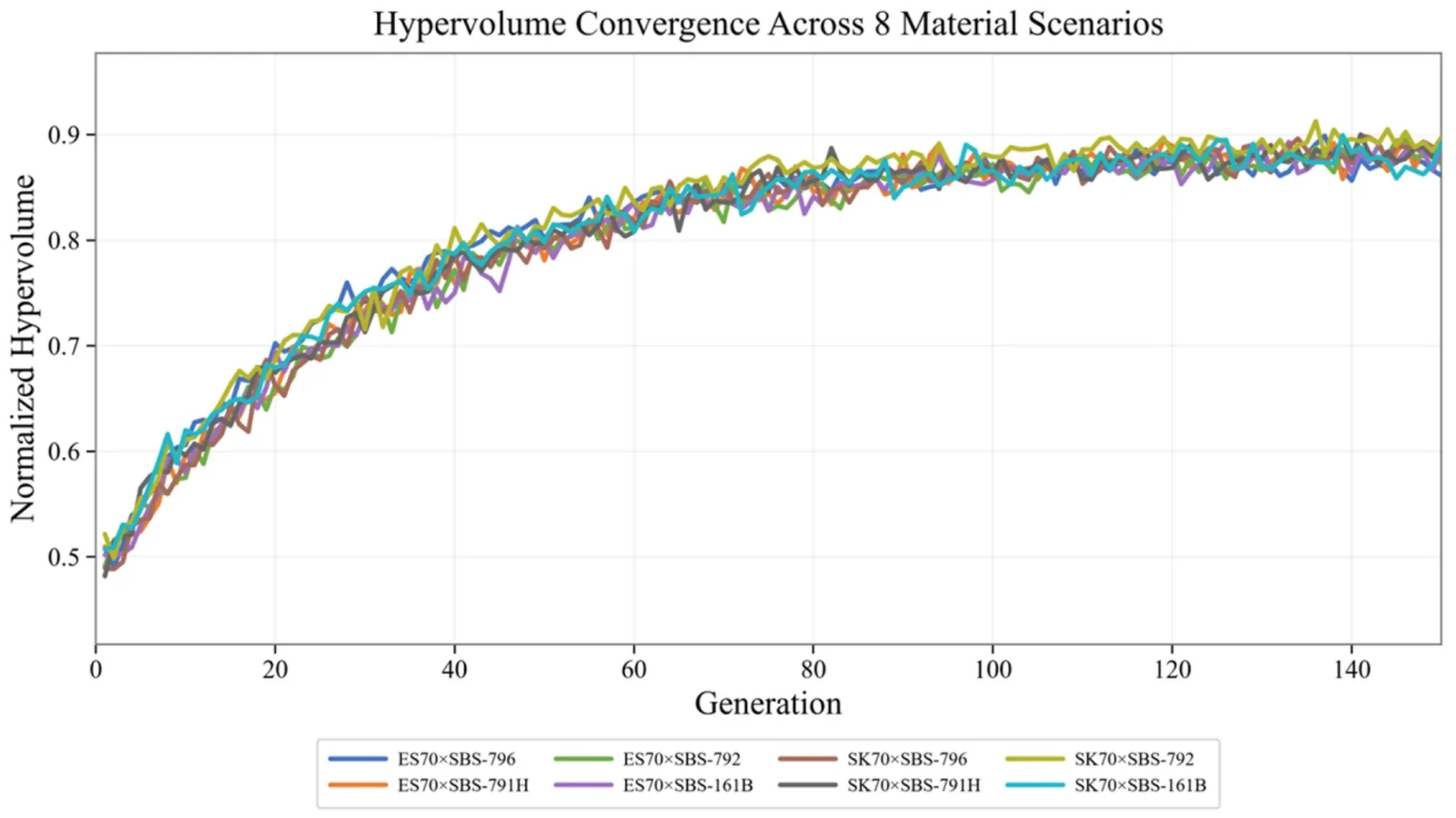
Convergence curve of NSGA-II showing hypervolume versus generation. Rapid early improvement is followed by gradual stabilization toward the end of the 150-generation search.

The hypervolume indicators increased rapidly during the early generations and then gradually approached stable levels, as shown in Figure 3. Similar convergence trends were observed for all eight material scenarios, although minor differences remained among the individual trajectories. The largest improvement occurred mainly during the early and intermediate stages of the search, followed by progressively smaller changes. By the final part of the 150-generation runs, the hypervolume values fluctuated only slightly around stable levels, indicating that further generations were unlikely to materially alter the scenario-specific non-dominated sets. This convergence behavior supports the use of 150 generations for each material scenario.

#### 2.2.2. Pareto Front and Trade-Offs

**Figure 4:**
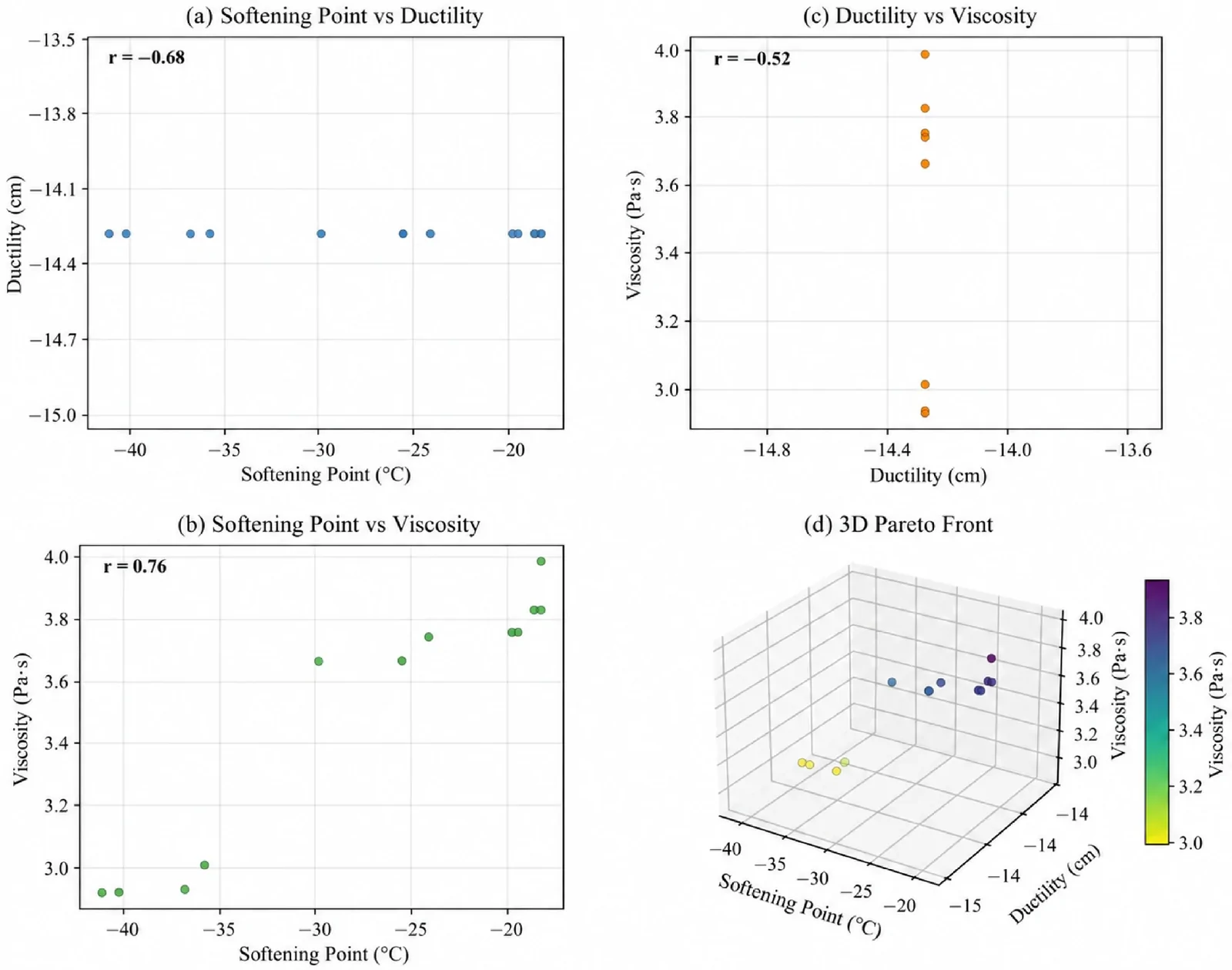
Trade-off matrix and three-dimensional Pareto representation in NSGA-II minimization coordinates. Softening point and ductility are plotted with negative signs because the two maximization objectives were converted to minimization form; viscosity is plotted directly. Each point represents a non-dominated candidate.

The pairwise relationships and three-dimensional representation of the three NSGA-II objectives are shown in Figure 4. Because NSGA-II was implemented as a minimization problem, the plotted optimization coordinates use softening point, ductility, and viscosity. The negative signs in the first two coordinates are therefore an algorithmic transformation rather than negative physical material properties. Reversing those signs for physical interpretation shows the expected trade-off structure: solutions with greater softening point generally require higher viscosity, while increases in softening point can be accompanied by reductions in ductility. The scatter matrix also shows that no single candidate simultaneously dominates all three physical objectives.

The NSGA-II optimization was conducted separately for each of the eight material scenarios defined by the combination of base asphalt type and SBS type. After 150 generations in each scenario, the scenario-specific non-dominated sets were pooled and filtered again by global dominance. This procedure produced 404 globally non-dominated candidates before the engineering reporting screen. The number confirms that the problem does not collapse to a narrow single optimum. Different material and processing combinations remain competitive because they exchange gains in softening point and ductility against viscosity and consistency.

**Figure 5:**
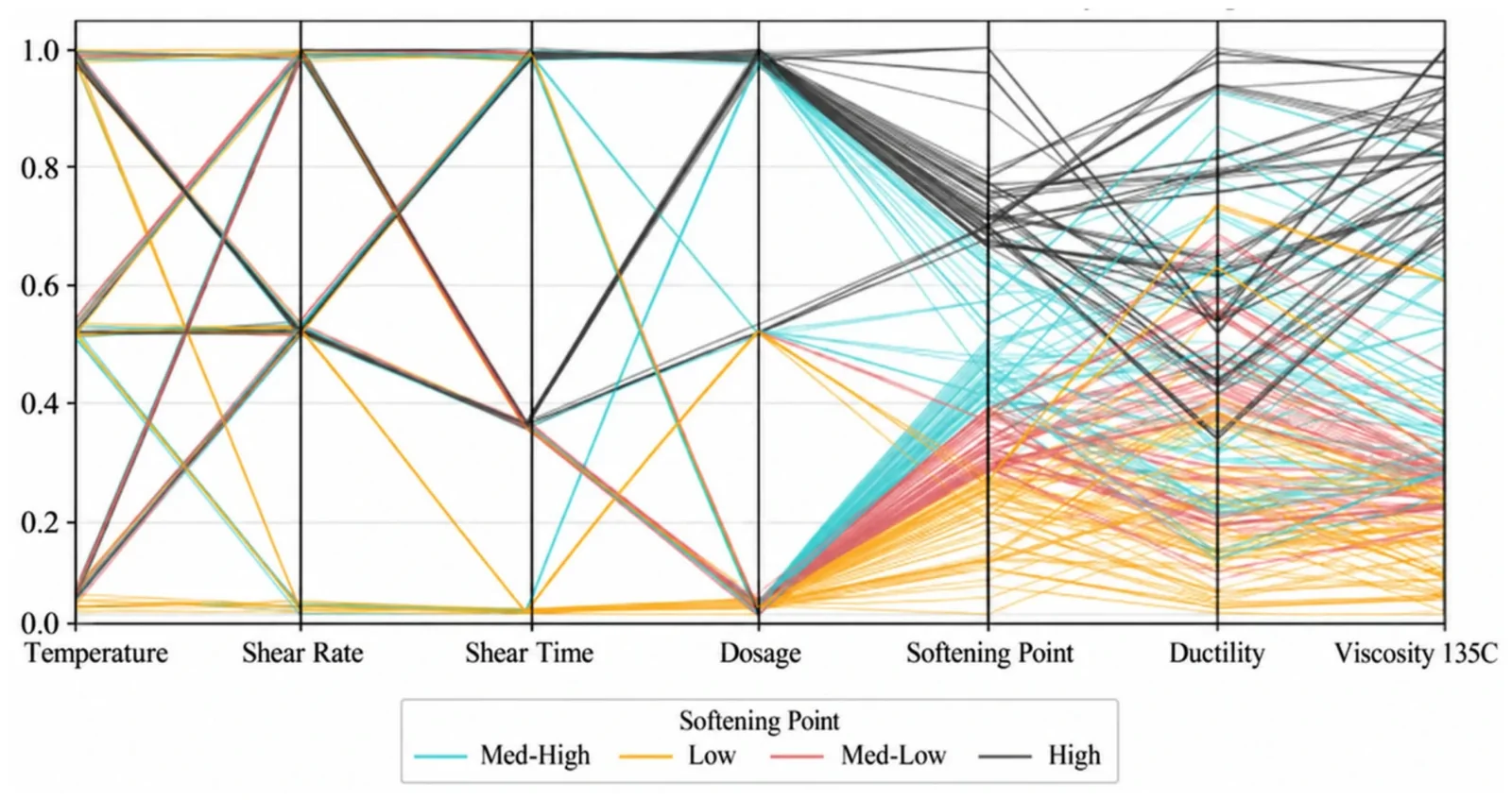
Parallel-coordinates representation of the 244 engineering-screened Pareto solutions, colored by softening-point level. All axes are min-max normalized to highlight the simultaneous distribution of the four controllable variables and the three response indicators within the retained Pareto set.

Applying the reporting screen of penetration between 45 and 70 (0.1 mm) and viscosity no greater than 3.0 Pa·s retained 244 candidates. These points are referred to as the screened Pareto set in the remainder of the results. The screen removes extreme high-viscosity solutions while preserving a broad range of softening point and ductility. It also eliminates very hard or very soft predictions outside the central penetration window. Because the screen is applied after the Pareto search, it does not bias the evolutionary process toward a prescribed property threshold. It simply focuses the engineering interpretation on solutions more likely to be relevant for follow-up testing. The structure of these 244 retained solutions is further visualized in the parallel-coordinates plot (Figure 5), which simultaneously displays the four controllable variables and the three key response indicators after min-max normalization.

Figure 5 further clarifies how the screened Pareto set spans the original design space. Shear temperature is concentrated near the upper-middle portion of its feasible range, whereas shear rate and shear time remain broadly distributed, indicating that multiple compensating processing pathways can lead to Pareto-efficient performance. The plot also shows that high softening-point solutions generally cluster with higher normalized softening point values but may correspond to a wide spread in ductility and viscosity, which reinforces the trade-off interpretation obtained from Figure 4.

The contribution of the eight material scenarios to the screened global front was uneven. ES70 with SBS-161B contributed 56 solutions, ES70 with SBS-791H and ES70 with SBS-796 each contributed 48, SK70 with SBS-161B contributed 45, SK70 with SBS-791H contributed 28, and SK70 with SBS-796 contributed 19. No SBS-792 solution survived the final screened global dominance comparison. This result should not be interpreted as a universal ranking of SBS products. It is specific to the supplied database, the three selected objectives, the reporting screen, and the explored processing ranges. SBS-792 may remain attractive under other objectives such as storage stability, aging resistance, cost, or different binder sources.

The absence of a single dominant material scenario is practically useful. It shows that process optimization is not equivalent to choosing one SBS type and then maximizing shear severity. Several combinations occupy distinct regions of the front. The scenario-wise formulation also prevents the algorithm from creating numerically convenient but physically meaningless mixtures between SBS categories.

### 2.3. Process Windows and Representative Designs

#### 2.3.1. Distribution of Controllable Variables on the Screened Pareto Set

The screened Pareto solutions occupy a restricted but not singular portion of the original process domain. Table 2 reports the median, interquartile range, and observed Pareto range for each continuous variable. SBS dosage has a median of 3.47%, with the middle 50% of solutions extending from 3.11% to 5.49%. The full screened range reaches approximately 6.35%, which indicates that higher dosages can be competitive for performance-oriented points but are not necessary for every Pareto-efficient design.

Shear temperature is more concentrated than the other process variables. The median is 181.14 °C and the interquartile range is 179.23 to 185.03 °C. Most efficient designs therefore lie near the middle to upper portion of the experimentally represented temperature domain. This pattern is consistent with the need to reduce binder viscosity sufficiently for polymer swelling and dispersion without treating the maximum temperature as automatically optimal.

Shear rate and shear time show much broader distributions. The rate interquartile range extends from about 2047 to 5822 rpm, while the time interquartile range extends from about 31.5 to 112.6 min. These wide spans indicate that several compensation mechanisms are possible. A relatively low shear rate can remain efficient in some material scenarios when other variables are favorable, while high rates can reduce the need for long treatment in other regions of the front. Consequently, it would be misleading to report one narrow universal window for rate or time. The Pareto set instead supports scenario-dependent recipes.

The distributions also illustrate why simple median-based process selection is not equivalent to optimization. Median values describe where data were collected, whereas Pareto distributions identify where efficient objective combinations occur according to the learned response surface. The two concepts should not be conflated.

Table 2 summarizes the distribution of dosage, temperature, shear rate, and shear time across the screened Pareto set. The statistics show that shear temperature is comparatively concentrated, whereas shear rate and shear time span broad intervals. SBS dosage also occupies a broad, non-uniform range. These results support the interpretation that Pareto efficiency can be reached through multiple compensating process combinations rather than through one universal operating window.

#### 2.3.2. Representative Pareto Designs

Four representative candidates are listed in Table 3. The balanced solution is the screened Pareto point with the minimum normalized distance to the ideal objective vector. It corresponds to ES70 base asphalt with SBS-161B. The process uses 3.24% SBS, a temperature of 179.1 °C, a rate of approximately 4796 rpm, and a time of 53.2 min. The DNN surrogate predicts a penetration of 54.3 (0.1 mm), softening point of 88.4 °C, ductility of 28.5 cm, and viscosity of 1.60 Pa·s. This point is not the maximum of any one objective. Its value is the balanced property profile obtained without imposing subjective objective weights during the search.

The high-softening-point candidate uses SK70 with SBS-161B at a much higher dosage of 5.83% and a long shear time of 113.4 min. Its predicted softening point reaches 129.1 °C, but viscosity rises to 2.90 Pa·s and penetration decreases to 46.1. Ductility remains 26.0 cm. This point lies near the intensive-modification side of the screened front and would be relevant only when high-temperature resistance is prioritized strongly enough to accept the workability penalty.

The high-ductility candidate uses ES70 with SBS-796 at 5.91% SBS, 178.8 °C, 5861 rpm, and 69.3 min. Its predicted ductility is 37.0 cm, accompanied by a softening point of 111.3 °C and viscosity of 2.77 Pa·s. This combination confirms that high softening point and high ductility can coexist in the learned domain, but the cost is a considerably higher viscosity than the balanced solution.

The low-viscosity candidate lies at the opposite edge of the front. It uses SK70 with SBS-791H at 3.0% dosage, nearly 190 °C, approximately 2000 rpm, and 30 min. The predicted viscosity is only 0.474 Pa·s, but softening point and ductility fall to 55.1 °C and 12.3 cm. This extreme demonstrates why minimizing viscosity alone is not a meaningful design objective. The multi-objective framework retains such a point because it is non-dominated in one direction, while the engineering decision can reject it if the associated performance is insufficient for a specific application.

These representative solutions should therefore be read as design alternatives, not as a rank ordering. The balanced point provides a starting recommendation for general-purpose applications. The three extreme points show the amount of property change available when one objective is emphasized. In an applied project, additional constraints such as climatic grade, cost, storage stability, aging resistance, or plant energy consumption could be imposed before a final recipe is selected.

### 2.4. Comparison with Median Baseline Recipe

To provide a reference for comparing the DNN-predicted performance of the optimized solution, a median baseline recipe was defined as the median values of the four processing parameters from the training set. This median baseline recipe represents the central tendency of the experimental conditions in the supplied database and is used here as a statistical benchmark, not as a claim of established industrial practice. The recipe consisted of shear temperature at 180 °C, shear rate at 4000 rpm, shear time at 60 min, and SBS dosage at 4.5%. The base asphalt type and SBS type were set to ES70 and SBS-161B, respectively, to match the composition of the balanced solution. The DNN surrogate predicted the following performance for this median baseline recipe: penetration of 51.2 (0.1 mm), softening point of 79.9 °C, ductility of 24.6 cm, and viscosity at 135 °C of 1.42 Pa·s. These values are DNN predictions, not experimental measurements.

The balanced solution from Table 3, in contrast, was predicted by the DNN to achieve a softening point of 88.4 °C, ductility of 28.5 cm, and viscosity of 1.60 Pa·s. Relative to the median baseline recipe, the DNN-predicted softening point of the balanced solution was 10.6% higher, while the predicted ductility changed by 15.9%. The predicted viscosity increased by 12.7%. These percentage changes are differences between two sets of DNN predictions and should not be interpreted as experimentally measured improvements. The predicted viscosity increase is modest relative to the predicted gains in softening point and ductility, but it represents a workability cost that should be considered in practical selection.

**Figure 6:**
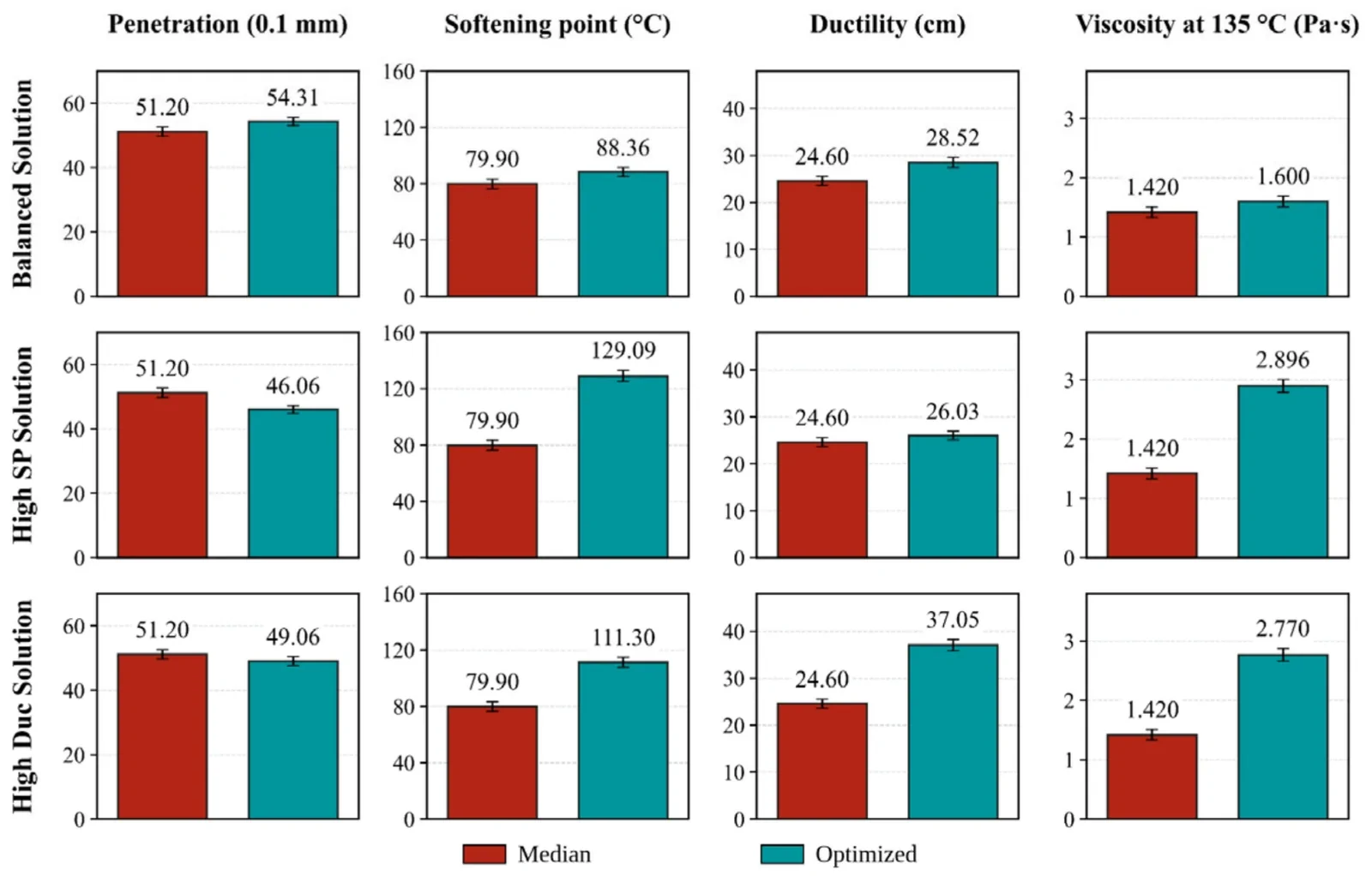
DNN-predicted physical-property comparison between the common median baseline recipe and the balanced, high-softening-point, and high-ductility representative Pareto solutions.

Figure 6 compares the DNN-predicted physical properties of the three representative Pareto solutions with a common median reference. The same median reference is retained in all three comparisons to provide a consistent statistical baseline. Relative to the median reference, the balanced solution shows simultaneous increases in softening point and ductility with a moderate increase in viscosity. The high-softening-point solution provides the largest increase in softening point, accompanied by lower penetration and substantially higher viscosity. In contrast, the high-ductility solution achieves the highest ductility among the three representative solutions while also exhibiting increased softening point and viscosity. These contrasting performance profiles illustrate the trade-offs among high-temperature performance, deformation capacity, and viscosity captured by the Pareto optimization.

### 2.5. Validation of Optimal Solutions

To confirm that the DNN surrogate predictions generalize to the optimized regions of the design space, an additional sample was prepared using the recommended parameters from the balanced solution listed in Table 3: ES70 base asphalt with SBS-161B, SBS dosage of 3.24%, shear temperature of 179.1 °C, shear rate of 4796 rpm, and shear time of 53.2 min. The sample was prepared following the same experimental protocols described in Section 4.2.1, and its properties were measured using the standard test methods outlined in Section 4.2.2.

The measured values were 53.8 (0.1 mm) for penetration, 87.6 °C for softening point, 27.9 cm for ductility, and 1.57 Pa·s for viscosity. Relative to the DNN predictions of 54.31, 88.36 °C, 28.52 cm, and 1.600 Pa·s, the corresponding relative errors were 0.95%, 0.87%, 2.22%, and 1.91%, respectively. Thus, all four measured properties agreed with the surrogate predictions within approximately 2.3%, supporting the physical achievability of the balanced solution.

**Figure 7:**
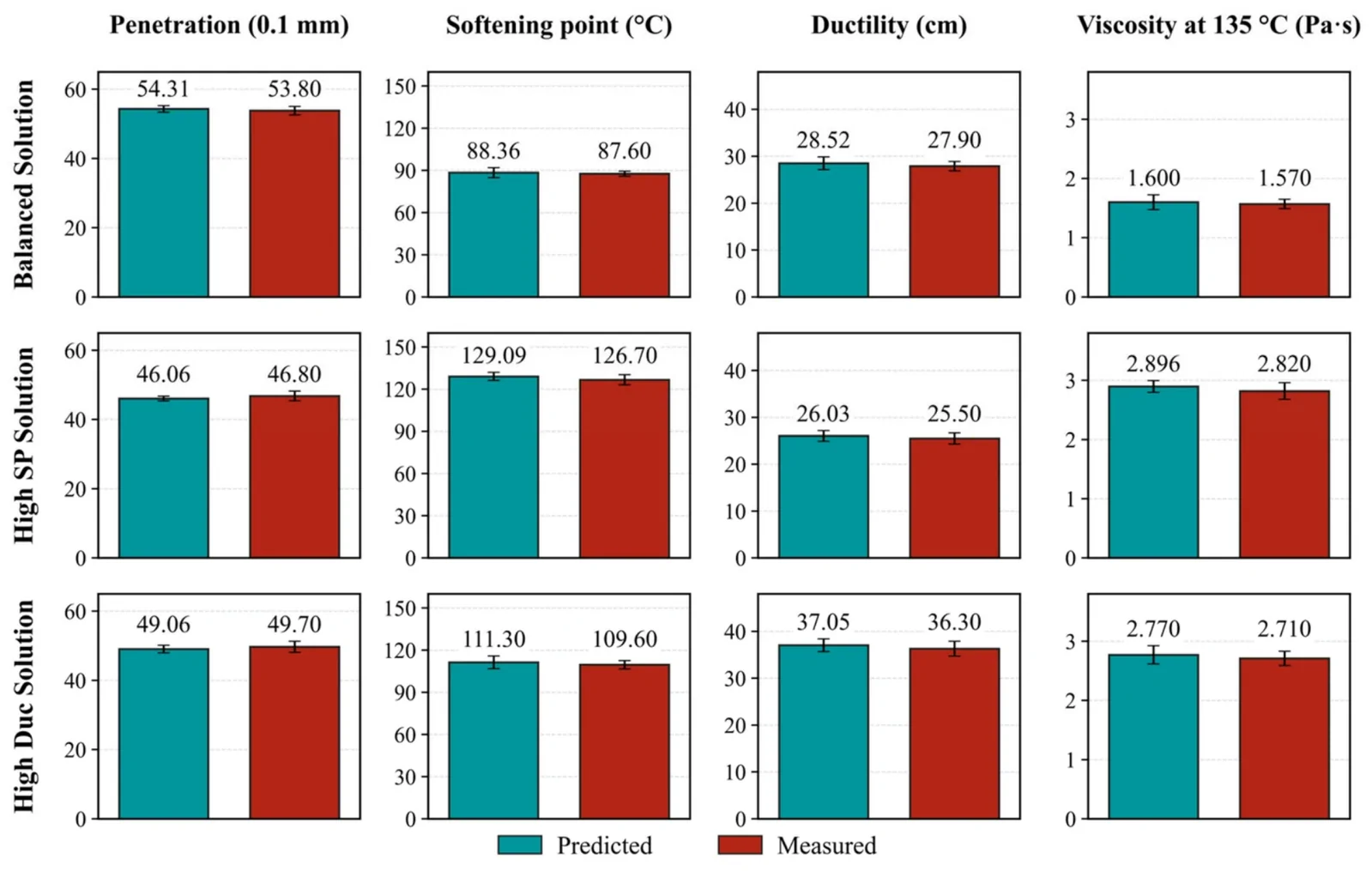
Predicted versus measured performance of the three representative Pareto solutions for penetration, softening point, ductility, and viscosity at 135 °C. Each condition was tested ten times; the reported values are averages, and the error bars represent the standard deviation of the ten replicate measurements. Relative deviations are calculated from the displayed predicted and measured values.

To further assess the robustness of the surrogate-assisted optimization across different regions represented by the Pareto set, the experimental validation was extended from the balanced solution to two additional representative solutions, namely the high-softening-point and high-ductility solutions. For each of the three representative conditions, ten independent replicate samples were prepared and tested. The values reported below and in Figure 7 are the averages of these ten replicate measurements, and the error bars represent the standard deviation of the ten replicates.

Figure 7 compares the DNN-predicted and experimentally measured values of penetration, softening point, ductility, and viscosity at 135 °C for all three representative solutions. For the balanced solution, the predicted and measured values were 54.31 and 53.80 (0.1 mm) for penetration, 88.36 and 87.60 °C for softening point, 28.52 and 27.90 cm for ductility, and 1.600 and 1.570 Pa·s for viscosity at 135 °C, respectively. For the high-softening-point solution, the predicted softening point of 129.09 °C was experimentally measured as 126.70 °C. The corresponding penetration, ductility, and viscosity also showed close agreement between prediction and experiment. Similarly, the high-ductility solution exhibited good agreement between the predicted and measured values for all four target properties.

Across the three experimentally tested representative Pareto solutions, the relative deviations between the DNN predictions and measured values ranged from approximately 0.86% to 2.62%. The largest deviation was observed for the viscosity of the high-softening-point solution and remained below 3%. These results provide additional experimental support for the physical feasibility and predictive consistency of the selected representative solutions. The validation evidence is limited to the three experimentally tested conditions and should not be interpreted as experimental verification of the entire Pareto front.

### 2.6. Response Surface Analysis

**Figure 8:**
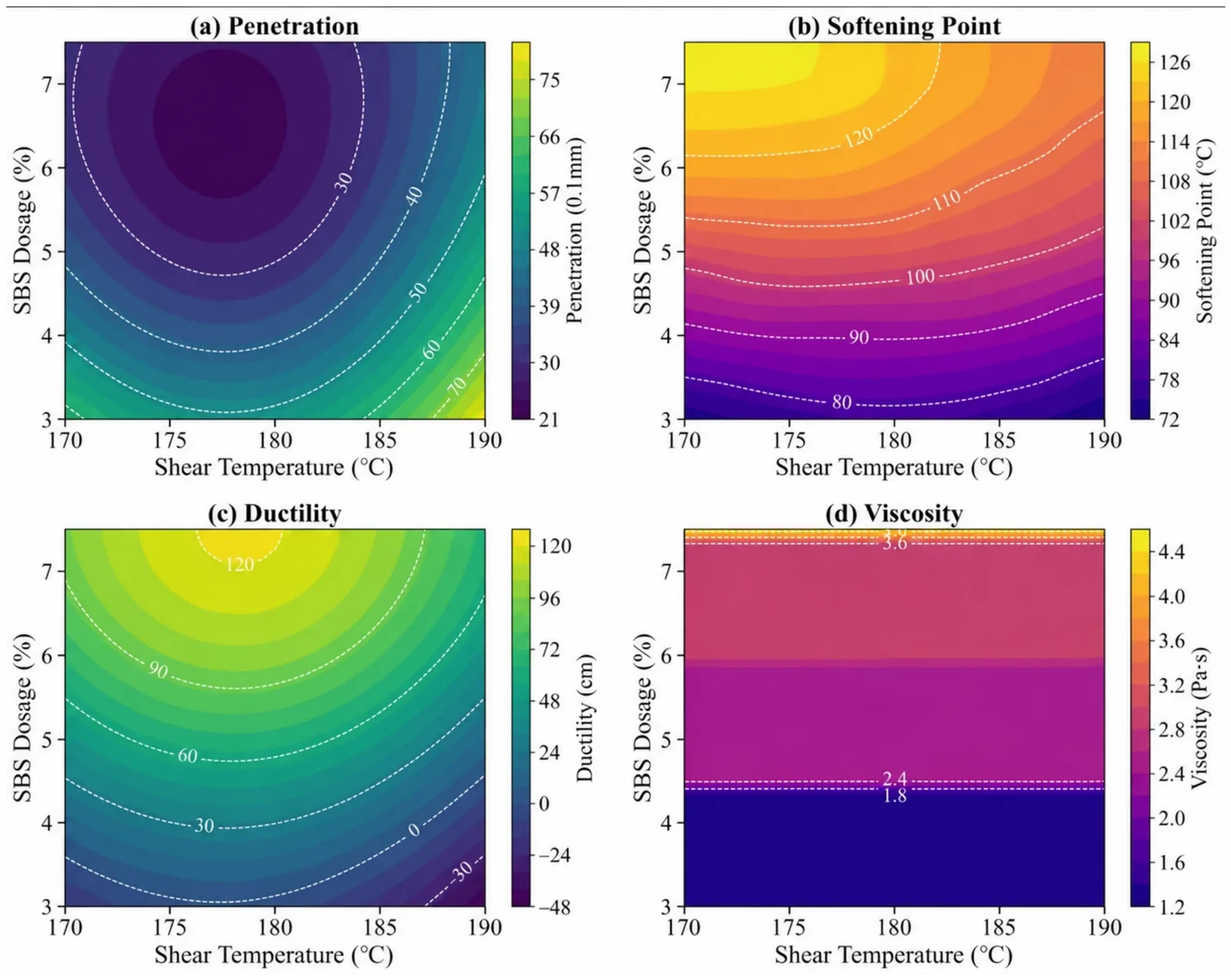
DNN response surfaces of (**a**) penetration, (**b**) softening point, (**c**) ductility, and (**d**) viscosity at 135 °C versus shear temperature and SBS dosage, with shear rate fixed at 4000 rpm and shear time fixed at 60 min.

Figure 8 shows DNN response surfaces for all four target properties as functions of shear temperature and SBS dosage, with shear rate fixed at 4000 rpm and shear time fixed at 60 min. The softening-point surface rises strongly with dosage and shows an additional temperature effect, whereas penetration shows a non-monotonic response across the same plane. Ductility changes markedly with dosage and temperature, and viscosity is dominated by dosage in the plotted slice. Taken together, the four surfaces illustrate why a single-variable rule cannot represent the process–performance relationship.

For softening point specifically, values above approximately 85 °C occur over a broad region at moderate-to-high SBS dosage, with the detailed boundary depending on temperature. At high dosage, further changes in temperature produce smaller incremental gains, indicating a local plateau. The other response surfaces show that moving toward this high-softening-point region also changes penetration, ductility, and viscosity, reinforcing the need for multi-objective rather than single-response optimization.

### 2.7. Microstructural Interpretation of Optimal Solutions

**Figure 9:**
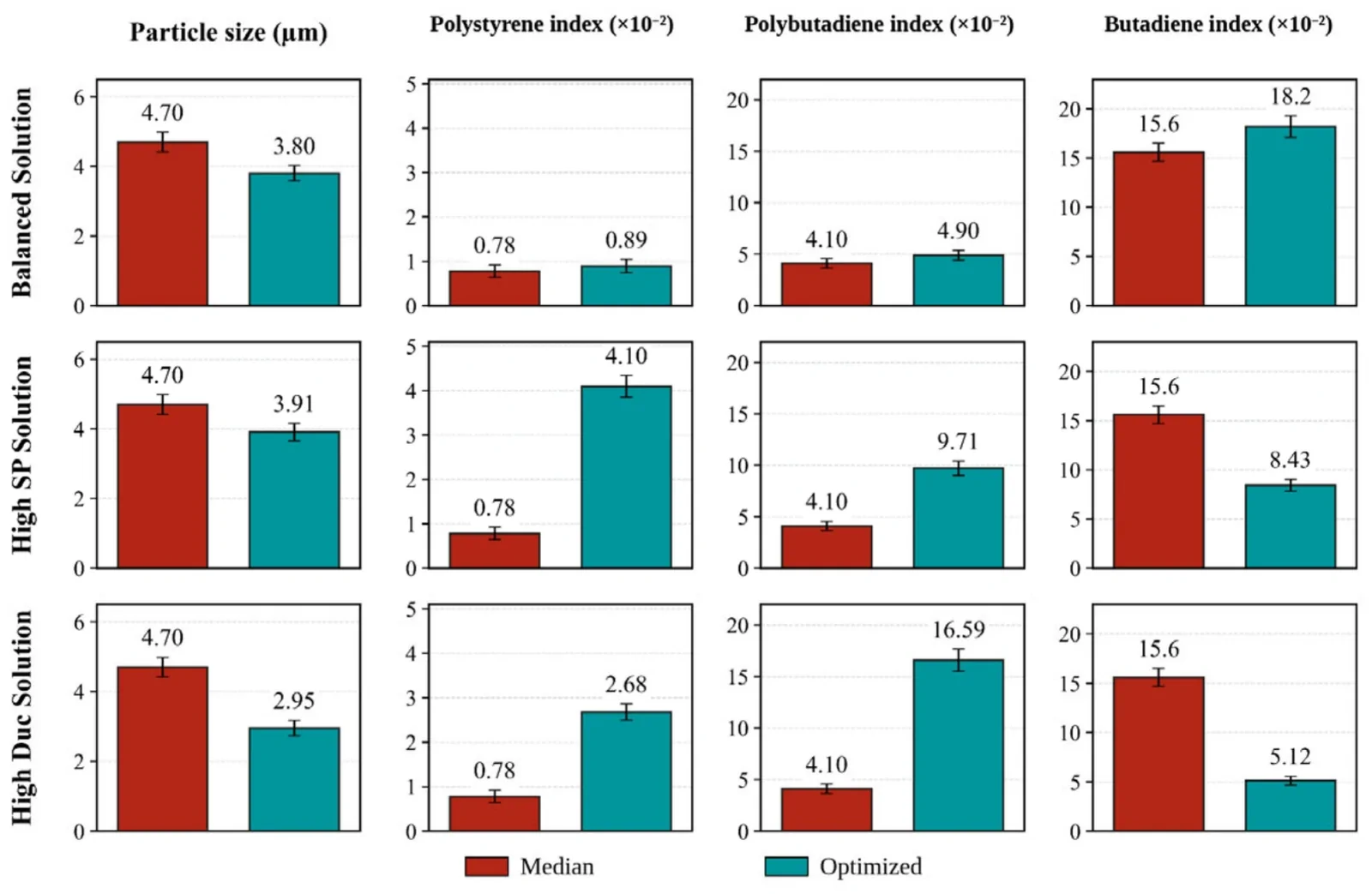
Comparison of microstructural descriptors between the common median baseline and three representative Pareto solutions: balanced solution, high-softening-point solution, and high-ductility solution. The descriptors include particle size, polystyrene index, polybutadiene index, and butadiene index.

Figure 9 compares the particle size and three FTIR-derived indices associated with the balanced, high-softening-point, and high-ductility solutions against the common median reference. The microstructural descriptors of the representative solutions were obtained from the experimental database within the corresponding base-asphalt/SBS system and processing space. For graphical clarity, the three FTIR-derived indices are displayed at 10^−2^ of their original database values. The same median reference is retained in all three comparisons to provide a consistent statistical baseline. Distinct microstructural characteristics are observed among the three representative solutions. The balanced solution exhibits a smaller particle size than the median reference, accompanied by moderate changes in the three FTIR-derived indices. The high-softening-point solution shows pronounced increases in the polystyrene and polybutadiene indices, while its butadiene index decreases relative to the median reference. In contrast, the high-ductility solution presents the smallest particle size and the largest polybutadiene index among the three representative solutions, together with a substantially lower butadiene index. These differences demonstrate that the representative Pareto solutions correspond to distinguishable combinations of particle dispersion and polymer-related spectral characteristics.

### 2.8. Radar Chart of Typical Solutions

**Figure 10:**
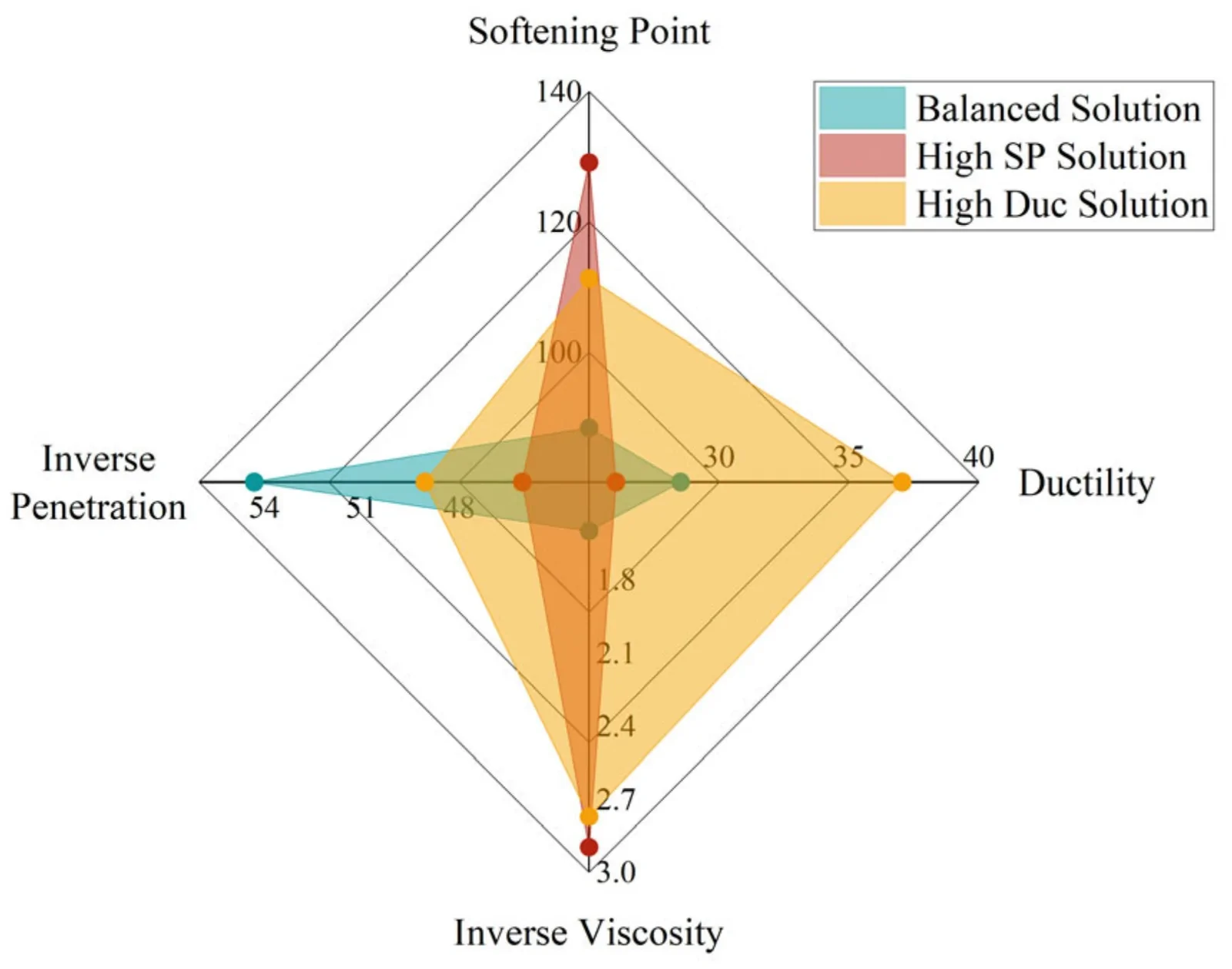
Radar chart comparing the three representative Pareto-optimal solutions: balanced solution, high-softening-point solution, and high-ductility solution. The four axes represent normalized softening point, ductility, inverse viscosity, and inverse penetration.

Figure 10 compares the three representative Pareto solutions using normalized softening point, ductility, inverse viscosity, and inverse penetration. The inverse quantities are used only to place indicators with opposite numerical directions on a common visual scale and should not be interpreted as independent “higher-is-better” performance criteria. In particular, lower penetration does not necessarily indicate superior overall binder performance and should be interpreted together with the other engineering properties. The radar chart is therefore used only to visualize differences in the performance profiles of the representative solutions rather than to provide an overall ranking based on polygon area. The high-softening-point solution shows the highest softening point but also the highest viscosity among the three representative designs, while the high-ductility solution emphasizes ductility. The balanced solution provides a more even profile across the considered indicators.

### 2.9. Schematic of Gel Network Evolution

**Figure 11:**
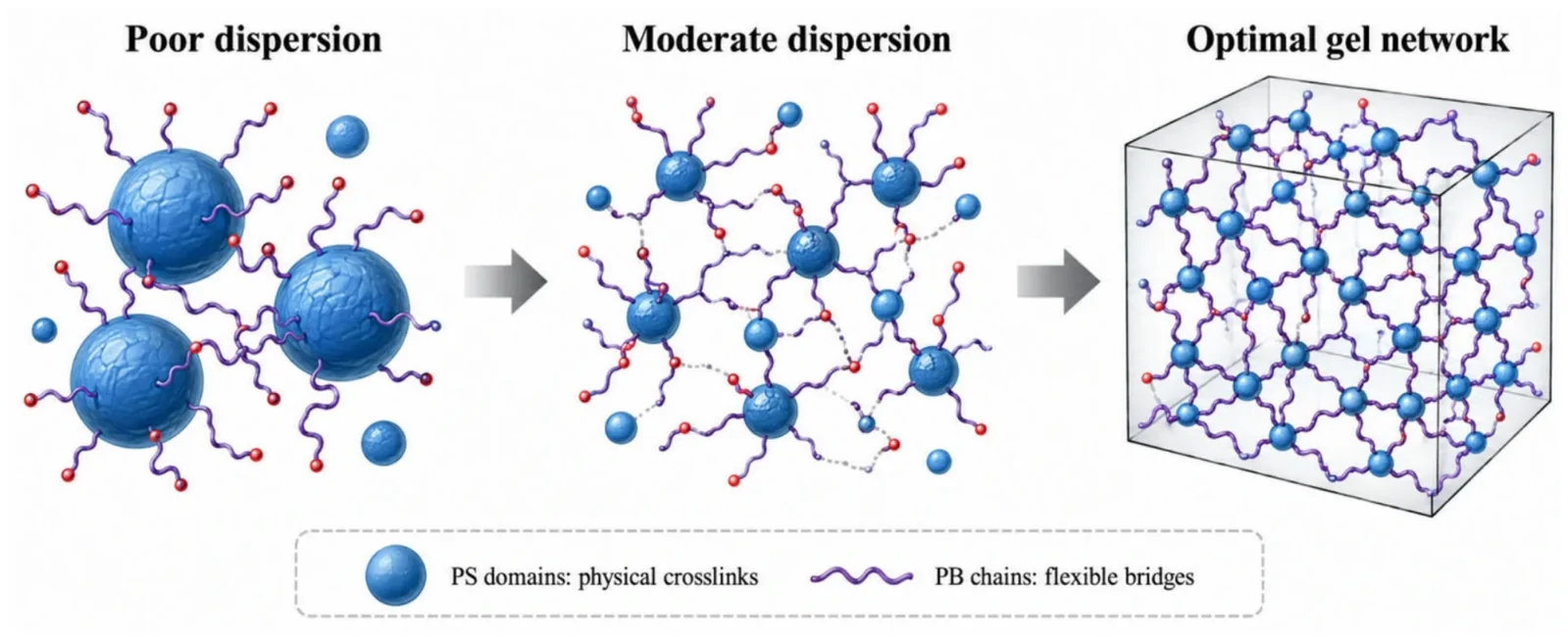
Conceptual schematic of possible SBS physical gel network evolution from poor dispersion to a more interconnected network. PS-rich domains act as physical crosslinking nodes, while PB chains provide flexible bridges between domains. The schematic is intended only as a conceptual interpretation of the associations observed in the dataset and of mechanisms reported in previous SBS literature.

Figure 11 presents a conceptual schematic of possible gel network evolution under different processing conditions. At low shear temperature and low shear rate, SBS particles may remain large and poorly dispersed, and the network may be discontinuous with many defects, which is consistent with the lower softening point and limited ductility observed in such regions. At moderate conditions, the particles may be broken down and more uniformly dispersed, forming a more continuous network with fewer defects. At the processing conditions associated with the balanced solution, the schematic suggests that SBS particles may be finely and uniformly dispersed, forming a more homogeneous network with well-developed polystyrene domains and flexible polybutadiene bridges. Such a network is consistent with the simultaneous improvement in softening point and ductility. However, the schematic is a conceptual interpretation based on established SBS modification mechanisms and the observed property trends; it is not direct microscopic evidence of the optimized sample and does not prove that the optimization itself generated a specific network morphology.

### 2.10. Physical Consistency of Optimization Results

The optimization results are consistent with established theory on SBS-modified asphalt gels, although this consistency should be regarded as a plausibility check rather than a direct mechanistic validation. The positive correlation between softening point and viscosity arises because both properties depend on the density and integrity of the SBS network. A denser network with more crosslinks resists deformation at high temperatures, raising the softening point. The same network increases internal friction, raising the viscosity. The negative correlation between softening point and ductility reflects the opposing requirements of network density and segmental mobility. High crosslink density restricts the movement of polybutadiene segments, which is beneficial for high-temperature performance but detrimental to low-temperature elongation. The trade-offs captured by the Pareto front are therefore physically plausible and consistent with the known behavior of SBS-modified binders, and they represent practical constraints in material design.

The balanced solution corresponds to a shear temperature of 179.1 °C, a shear rate of approximately 4796 rpm, a shear time of 53.2 min, and an SBS dosage of 3.24%. These values lie within the experimentally represented design domain. The result also illustrates that higher SBS dosage is not automatically optimal: in the learned response surface, a lower dosage can remain Pareto-efficient when compensated by an appropriate combination of shear rate, temperature, and time. These interpretations remain at the level of process-property associations; they do not constitute direct evidence of a specific microstructural mechanism.

### 2.11. Advantages of the Proposed Framework

#### 2.11.1. Advantages of the Single DNN Surrogate

The use of a single multi-output DNN surrogate offers several advantages over multiple independent models. First, the shared network provides all four physical-property predictions through one preprocessing pipeline and one inference call, reducing the risk of inconsistencies among separate surrogate families. Second, the computational burden of the evolutionary search remains small relative to laboratory testing. With 120 individuals, 150 generations, and eight material scenarios, the reported search requires on the order of 144,000 candidate evaluations, all performed by the same frozen DNN. Third, the single-network structure keeps the response representation compact and can support future extensions to alternative optimization strategies without changing the data interface.

These advantages make the DNN surrogate well-suited for the iterative evaluation demands of NSGA-II. The high accuracy reported in Section 2.1 confirms that the single-network approach does not sacrifice predictive performance for operational convenience. The optimization stage can therefore focus on exploring objective trade-offs instead of compensating for model inconsistencies.

#### 2.11.2. Comparison with Literature Data

**Figure 12:**
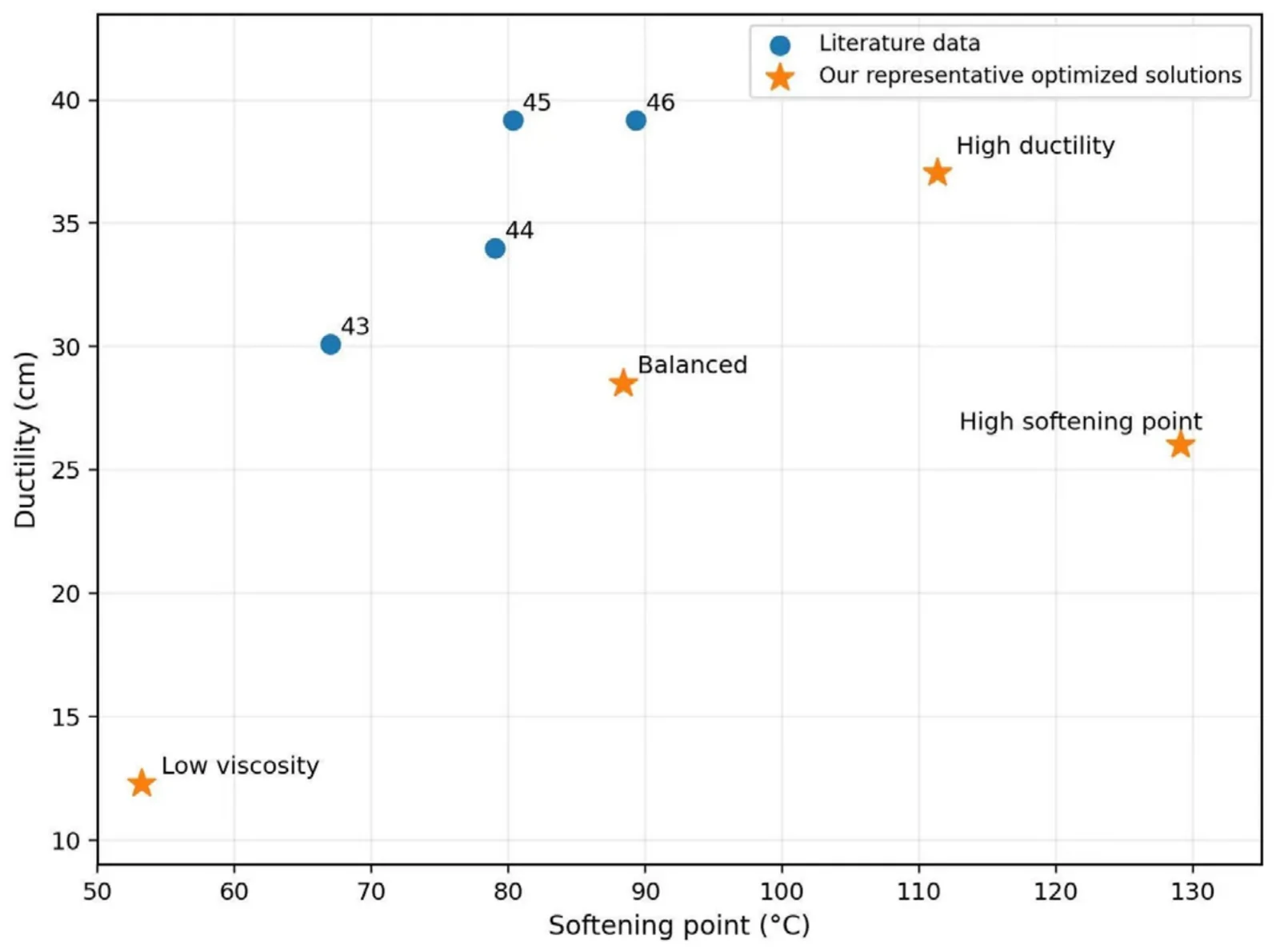
Literature benchmarking of softening point versus ductility for the four representative optimized solutions and selected SBS-based binder data from Refs. [43,44,45,46]. Literature values are plotted as reported in the source papers; differences in binder source, formulation, and test protocol should be considered when interpreting the comparison.

The optimized designs were benchmarked against representative SBS-based binders reported in the literature. To ensure comparability of the ductility results, only literature data measured at 5 °C with an extension rate of 5 cm/min were retained for comparison in Table 4 and Figure 12. Although differences in base asphalt and modifier formulation remain among the cited studies, the consistent ductility test conditions provide a common basis for comparison. The balanced solution (88.4 °C softening point and 28.5 cm ductility) lies toward the upper end of the literature softening-point range while maintaining moderate ductility. The high-softening-point and high-ductility representative solutions extend further into the high-performance region of the two-property space.

Figure 12 compares the four representative optimized designs from Table 3 with four published SBS-based binder data points [43,44,45,46]. The selected literature points span approximately 67.0–89.3 °C in softening point and 30.1–39.2 cm in ductility. The balanced solution remains within the broader published performance envelope, whereas the high-softening-point and high-ductility designs occupy regions with substantially higher softening point. This comparison supports the value of the optimization framework for exploring performance combinations beyond conventional central recipes, while also showing that the balanced solution is not an isolated or physically implausible prediction.

The external comparison should therefore be interpreted conservatively. It demonstrates that the surrogate-assisted search can identify a wider set of candidate property combinations than one median baseline recipe, but it does not by itself prove universal superiority over published SBS binders. The strongest evidence for the proposed framework remains the internally consistent Pareto search together with experimental confirmation of the balanced point.

The improvement over the traditional median recipe further demonstrates the value of systematic optimization. The median baseline recipe, representing the central tendency of the experimental conditions, was not optimal for any specific objective. The optimization framework identified a combination of parameters that improved both softening point and ductility with only a moderate increase in viscosity. This improvement is particularly valuable because it reduces the need for trade-off decisions in practice. Engineers can adopt the balanced solution to achieve better overall performance without sacrificing any single property substantially.

### 2.12. Limitations, Challenges, and Future Work

This section explicitly states the main challenges and limitations of the present study. These limitations define the scope within which the proposed DNN-NSGA-II framework should be interpreted and used:

(1) The microstructural descriptors were excluded from the optimization decision vector because they are not independently controllable production settings. They are used only for descriptive interpretation. A future extension should explicitly learn a process-to-microstructure mapping before microstructure is introduced as an intermediate design variable or mechanistic constraint.

(2) The optimization considered only three objectives: softening point, ductility, and viscosity. Additional objectives such as cost, energy consumption, and aging resistance could be included in a more comprehensive framework after the necessary data are available.

(3) The experimental validation was limited to three representative Pareto solutions: the balanced solution, the high-softening-point solution, and the high-ductility solution. Although these three conditions cover different regions of the Pareto set, the validation does not constitute experimental verification of the entire Pareto front.

(4) The dataset was limited to two base asphalt types (ES70 and SK70) and four SBS types (SBS-796, SBS-791H, SBS-792, and SBS-161B). The optimization results may not be directly transferable to other material systems. Future work could expand the dataset to include more modifier types, base asphalts, and aging conditions.

(5) Some extreme Pareto solutions, such as the high-softening-point candidate with a predicted softening point of 129.1 °C, lie beyond the literature range used for external benchmarking. These extreme predictions require dedicated experimental verification before they can be recommended for practical application.

(6) The database combines 756 records from a public experimental dataset and 108 in-house records. Although all records use consistent variable definitions and units, possible heterogeneity in source laboratories, equipment, and conditioning procedures remains a limitation that should be considered when interpreting the model accuracy.

(7) The model is applicable only within the investigated process ranges: SBS dosage 3.0–7.5%, shear temperature 170–190 °C, shear rate 2000–6000 rpm, and shear time 30–120 min. Extrapolation outside these ranges is not supported by the present data.

## 3. Conclusions

This study developed a surrogate-assisted multi-objective optimization framework for the inverse design of SBS-modified asphalt gels. A single multi-output DNN surrogate was trained on 864 experimental records and then coupled with NSGA-II to search for Pareto-optimal processing conditions. The main conclusions are summarized as follows.

(1) A single multi-output DNN surrogate trained on 864 complete records accurately predicted penetration, softening point, ductility, and viscosity at 135 °C, with test-set R^2^ values above 0.99 for all four targets. The 604/130/130 training-validation-test split and the held-out test evaluation support the use of the DNN as a fast response surface within the bounded experimental domain.

(2) The NSGA-II algorithm generated a set of Pareto-optimal solutions that clearly illustrated the trade-offs among softening point, ductility, and viscosity. Softening point showed a strong positive correlation with viscosity and a negative correlation with ductility among the Pareto solutions. No single solution maximized all three objectives simultaneously; the preferred choice depended on the specific performance priorities of the application.

(3) The balanced solution was identified for ES70 with SBS-161B at 3.24% SBS, 179.1 °C, approximately 4796 rpm, and 53.2 min. The DNN predicted a softening point of 88.4 °C, ductility of 28.5 cm, and viscosity of 1.60 Pa·s. Relative to the median baseline recipe, the DNN-predicted softening point and ductility were 10.6% and 15.9% higher, respectively, while the predicted viscosity increased by 12.7%. These percentage changes are differences between two sets of DNN predictions and should not be interpreted as experimentally measured improvements.

(4) The experimental validation was extended from the balanced solution to two additional representative Pareto solutions, namely the high-softening-point and high-ductility solutions. Across the three tested conditions, the relative deviations between the DNN predictions and the measured values ranged from approximately 0.86% to 2.62%, with the largest deviation remaining below 3%. These results support the physical feasibility and predictive consistency of the selected representative solutions. The validation evidence is limited to the three experimentally tested conditions and does not constitute experimental verification of the entire Pareto front.

(5) The screened Pareto set did not collapse to one narrow process window. Shear temperature was comparatively concentrated, with a median of 181.14 °C and an interquartile range of 179.23–185.03 °C, whereas shear rate and shear time remained broadly distributed. SBS dosage had a median of 3.47% and an interquartile range of 3.11–5.49%. These distributions indicate that multiple compensating process combinations can be Pareto-efficient rather than a single universal recipe.

(6) The post hoc microstructural descriptors associated with the balanced recipe showed a smaller particle-size value and larger polymer-related FTIR indices than the median baseline recipe. These descriptors provide a mechanism-oriented context for the observed property trends, but they are not DNN outputs and were not optimized directly. The microstructural comparisons are strictly observational trends within the supplied dataset, and they do not establish a causal process-microstructure-property relationship.

(7) The proposed framework closes the loop from predictive modeling to processing recommendation within the investigated material and process domain. It provides a data-driven and efficient tool for rational process design of SBS-modified asphalt gels. The framework may be extended in future work to include additional objectives such as cost, energy consumption, and aging resistance, and may be adapted to other polymer gel systems after retraining and validation with appropriate data.

The model developed in this study is applicable only to the two base asphalts and four SBS types investigated here, within the process ranges of SBS dosage 3.0–7.5%, shear temperature 170–190 °C, shear rate 2000–6000 rpm, and shear time 30–120 min. It should not be generalized to other base asphalts, SBS types, or process conditions without retraining and validation. Future work should focus on expanding the dataset to include more modifier types and aging conditions, developing two-stage optimization with microstructure as intermediate variables, and implementing the framework in an industrial production setting.

## 4. Materials and Methods

### 4.1. Study Design and Data Domain

#### 4.1.1. Data Sources and Controllable Design Variables

The database used in this study contains 864 complete SBS-modified asphalt records arranged under a consistent set of 14 fields. The records span base asphalt identity, SBS type, dosage, three shear variables, four microstructural descriptors, and four performance labels. Of the 864 records, 756 (87.5%) were obtained from the Cement-Asphalt-Based Materials Public Dataset provided by Southwest Jiaotong University (https://nbsdc.cn/general/dataDetail?id=69d7cd46f175606608ea1e0f&type=1, accessed on 23 September 2026), and the remaining 108 (12.5%) were generated by our research group following the standard preparation and testing workflow. All 864 records represent experimental measurements; no simulated, synthetic, or interpolated data were included. The in-house measurements were incorporated using the same variable definitions, units, and target-label definitions as the public dataset. Only records with complete information in all 14 fields were retained.

During data compilation, replicate observations for the same material–process combination were identified by matching base asphalt, SBS type, dosage, shear temperature, shear rate, and shear time. Where replicates were present in the original source data, they were either averaged into a single representative value before inclusion in the 864-record database or, if they were retained as separate records, kept together as a group during the training/validation/test split so that the same material-process combination never appeared in more than one subset. This grouping prevents information leakage between model training and evaluation. The final dataset was divided into 604 training, 130 validation, and 130 test records randomly, and all scaling statistics were calculated from the training subset only. Because the modeling task in this paper is process recommendation, the variables were divided according to whether they can be specified before preparation. Base asphalt type, SBS type, SBS dosage, shear temperature, shear rate, and shear time were defined as design descriptors. Particle size and the FTIR indices $I_{PS}$, $I_{PB}$, and $I_{B}$ were treated as descriptive post-preparation features and were not provided to the DNN. The four measured performance indicators were used as target labels.

The resulting dataset exhibits a balanced factorial structure for the major categorical and process settings. ES70 and SK70 each contribute 432 records. Four SBS types, SBS-796, SBS-791H, SBS-792, and SBS-161B, each contribute 216 records. Each of the four dosage levels contributes 216 records, and each of the three levels of temperature, shear rate, and shear time is represented systematically across the material combinations. This structure is advantageous for learning interpolation within the observed range because it limits large empty regions in the factorial grid. It does not, however, justify extrapolation outside that range. All optimization bounds were therefore restricted to the minimum and maximum values observed in the database.

Table 5 summarizes the design domain and the statistical ranges of the descriptive variables and target labels. The continuous optimization domain is defined by dosage, temperature, rate, and time. Although the measured database contains discrete experimental levels for several of these variables, the DNN provides a continuous approximation inside the observed bounds. NSGA-II can therefore investigate intermediate settings that were not explicitly tested. Such interpolation is the main source of computational value in the proposed framework, but it also creates the need for later experimental confirmation of the selected continuous optimum.

#### 4.1.2. Target Labels and Descriptive Microstructure

**Figure 13:**
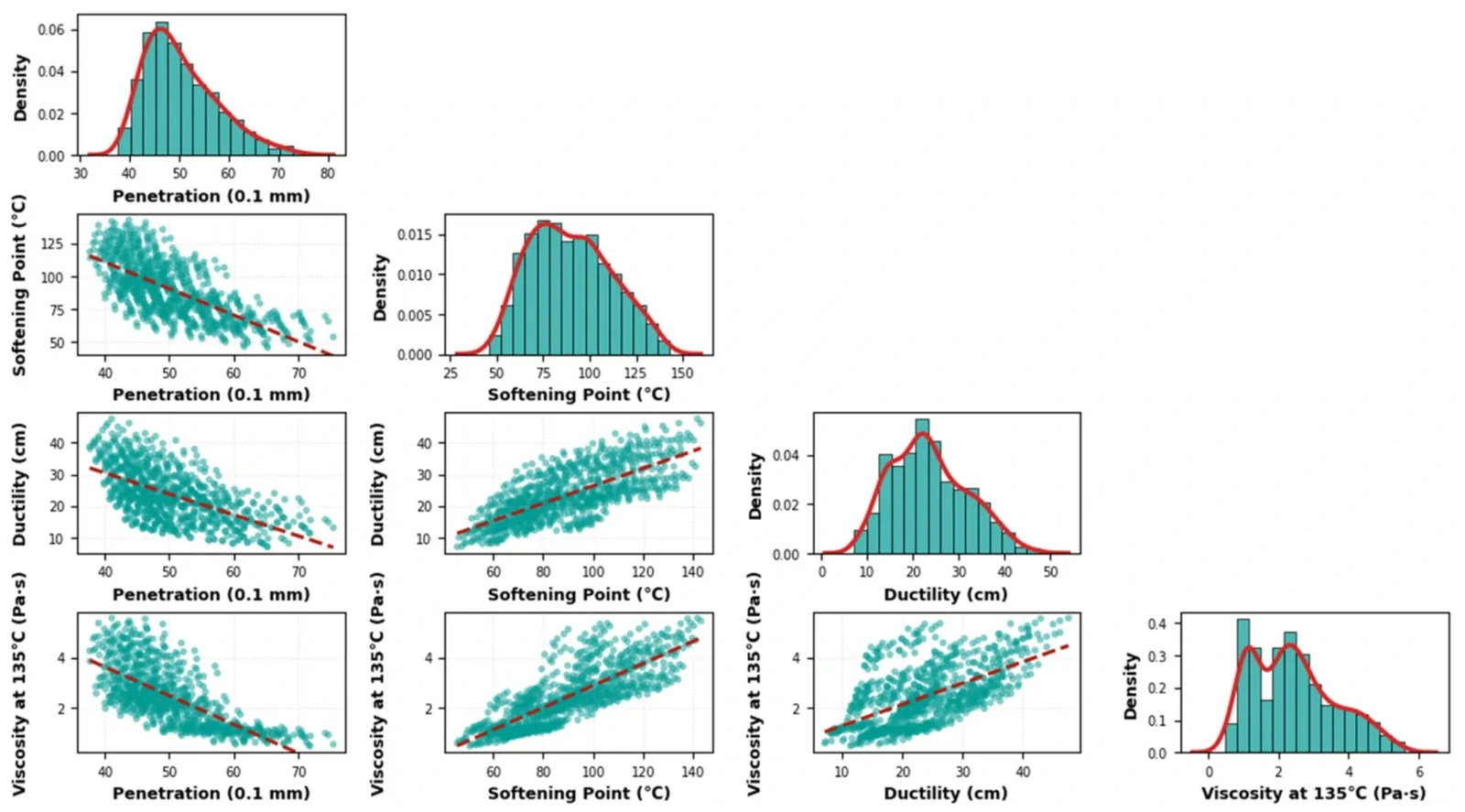
Correlation map and marginal distributions of the four performance labels in the 864-record database. The red dashed lines in the off-diagonal scatter plots indicate the fitted linear regression trends.

The four target labels were selected because together they describe complementary aspects of binder behavior that are directly relevant to process design. Penetration provides a conventional measure of consistency. Softening point reflects the temperature at which the binder reaches a prescribed level of softness under the test procedure and is frequently used as an empirical indicator of high-temperature resistance. Ductility describes extension capacity under standardized loading and conditioning. Rotational viscosity at 135 °C represents high-temperature flow resistance and is closely related to mixing, pumping, coating, and workability. The use of these four labels also allows the optimizer to expose practical conflicts. A recipe that strengthens the SBS network can increase softening point and sometimes ductility, yet the same structural development may raise viscosity and reduce penetration. The marginal distributions and pairwise relationships among the four performance labels are summarized in Figure 13.

The microstructural fields were retained because they provide important context for the database and for future mechanism-oriented work. Particle size reflects the degree of SBS breakup and dispersion. The FTIR-derived indices represent spectral information associated with the polymer phases and their relative signatures. These descriptors can be highly predictive when measured after preparation, which was one reason they were useful in our previous forward-modeling study [29]. In an inverse process-design setting, however, they cannot be treated as freely selectable inputs unless a second model explicitly links processing conditions to microstructure. The present paper avoids that causal shortcut. Its DNN is trained only on information that an engineer can choose before preparation, while the microstructure fields are used to describe the broader data domain and to motivate later extensions.

Before model construction, the pairwise correlations among the four target labels were examined as a descriptive check. Penetration was negatively correlated with softening point, ductility, and viscosity, with Pearson coefficients of −0.681, −0.587, and −0.704, respectively. Softening point was positively correlated with ductility at 0.725 and with viscosity at 0.804. Ductility and viscosity had a positive correlation of 0.591. These correlations are dataset-level tendencies rather than deterministic material laws. Their role is to show that the objectives are statistically coupled before optimization and that a multi-objective formulation is justified.

### 4.2. SBS-Modified Asphalt Preparation and Performance Testing

#### 4.2.1. Preparation Protocol Represented by the Database

**Figure 14:**
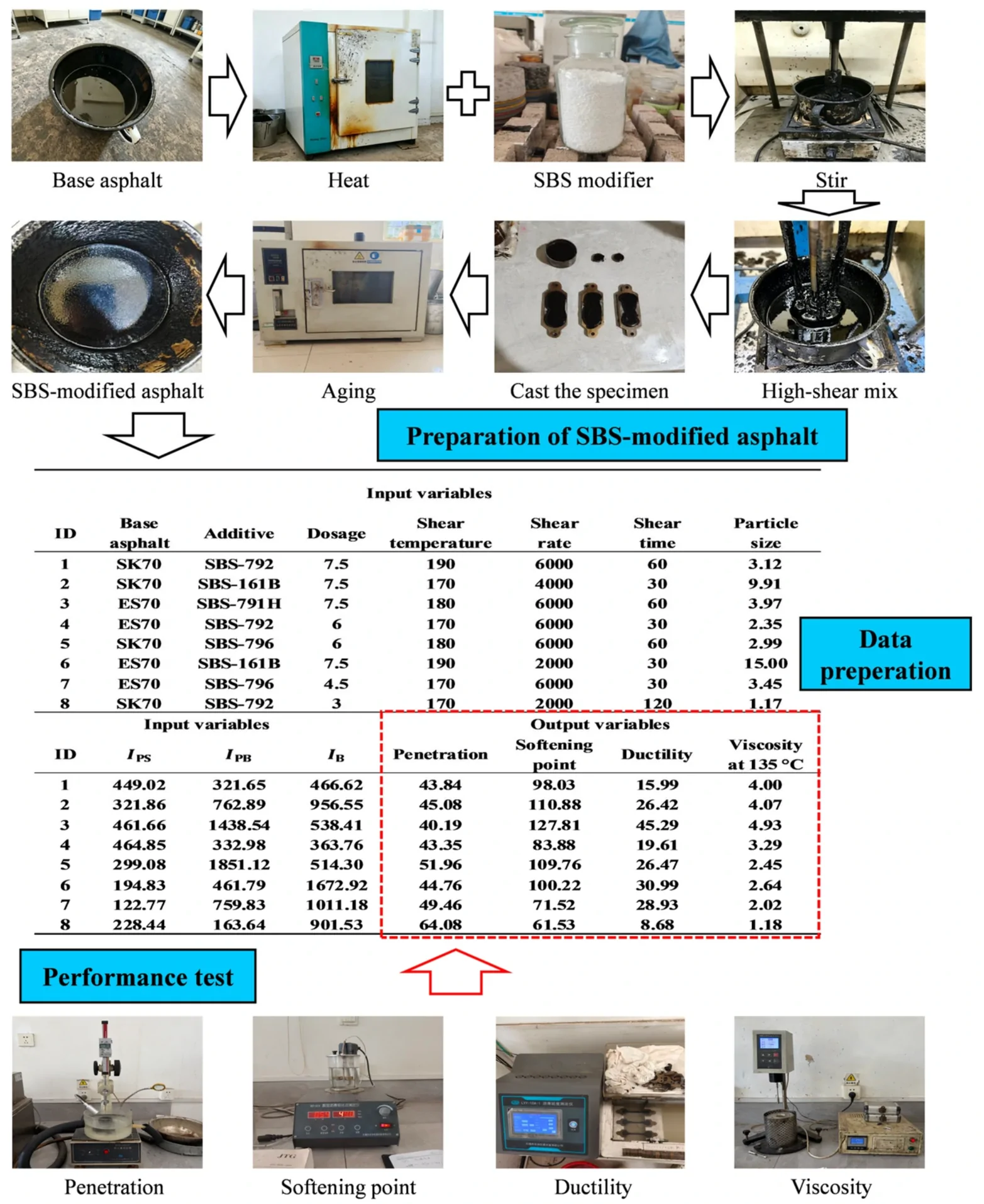
Schematic diagram illustrating the sample preparation, dataset construction, and performance-testing workflow for SBS-modified asphalt binders.

The complete experimental workflow for specimen manufacturing, data collation and laboratory performance measurement is summarized in Figure 14. The process variables represented in the database correspond to the main controllable stages of SBS-modified asphalt preparation. Base asphalt and SBS type define the material scenario. SBS dosage controls the polymer fraction. Shear temperature establishes the thermal condition for viscosity reduction, polymer swelling, and mass transfer between asphalt fractions and SBS. Shear rate controls the magnitude of mechanical input available to break agglomerates and distribute the modifier. Shear time determines the duration of thermal exposure and the extent to which dispersion and swelling can approach a stable state. The optimization bounds were chosen to coincide with the experimentally represented domain rather than with a broader nominal industrial range, because a data-driven surrogate should not be asked to extrapolate beyond the evidence on which it was trained.

For the subset generated by our research group, the preparation sequence followed the same variable definitions used throughout the database. ES70 and SK70 base asphalts, as well as SBS-796, SBS-791H, SBS-792, and SBS-161B, were all supplied by Yilu Hengfeng Hengshui Asphalt Technology Co., Ltd., Hengshui, Hebei, China. The base asphalt was heated to the selected processing temperature, the prescribed SBS dosage was added under controlled mixing, and high-shear processing was conducted at the target rotational speed and duration. Samples were then conditioned for the corresponding physical-property tests. For the in-house samples, the base asphalt was heated to the target shear temperature in a thermostatic oil bath. SBS was added at the prescribed dosage and pre-mixed at 500 rpm for 10 min. The blend was then sheared using a high-shear mixer at the 2000–6000 rpm rate for 30–120 min time. The temperature was controlled within ±2 °C. After shearing, the samples were conditioned at room temperature for 24 h and stored in sealed containers before testing. The important point for the present optimization framework is that dosage, temperature, rate, and time are actual controllable settings rather than latent features inferred after the sample has been produced.

The broad design range has practical value because it contains both mild and intensive processing conditions. At low dosage and low mechanical input, the polymer network may remain insufficiently developed. At high dosage and extended high-shear treatment, viscosity can rise substantially and polymer degradation may become a concern. The optimizer therefore searches for combinations rather than simply pushing every variable to its upper bound. This is one reason a population-based Pareto method is preferable to independent one-factor recommendations.

#### 4.2.2. Physical Performance Indicators and Testing

Penetration, softening point, ductility, and rotational viscosity were used as the physical performance outputs. These indicators are standard descriptors of asphalt binder consistency, thermal susceptibility, deformability, and workability. The testing logic follows commonly used procedures represented by ASTM D5/D5M for penetration [47], ASTM D36/D36M for softening point [48], ASTM D113 for ductility [49], and ASTM D4402/D4402M for rotational viscosity [50]. Ductility was measured at 5 °C with an extension rate of 5 cm/min. The viscosity label in the database is reported at 135 °C. Penetration tests were performed using a penetration apparatus (Model LYY-10A, Wuxi Petroleum Instrument Co., Ltd., Wuxi, China). Softening point was measured using a digital softening point tester (Model WSY-025B, Wuxi Petroleum Instrument Co., Ltd., Wuxi, China). Ductility was measured using a ductility testing machine (Model LYY-10-1, Wuxi Petroleum Instrument Co., Ltd., Wuxi, China). Rotational viscosity was measured using a rotational viscometer (Model NDJ-1C, Wuxi Petroleum Instrument Co., Ltd., Wuxi, China). For records compiled from different sources, unit harmonization and label consistency are essential, and only complete records with the same target definitions were retained in the supplied dataset.

The four indicators should not be interpreted as independent pass-fail criteria in this manuscript. Their primary role is to provide a multidimensional performance vector for the surrogate and optimizer. Penetration is predicted by the DNN and is later used as an engineering screening quantity. Softening point and ductility are treated as performance objectives to be increased. Viscosity is treated as an objective to be decreased because excessively viscous binders impose processing penalties even if high-temperature resistance is improved. This formulation reflects the practical need to balance performance and constructability without assuming that one universal recipe is optimal for all applications.

### 4.3. DNN Surrogate Construction

#### 4.3.1. Input Encoding, Scaling, and Data Split

A single multi-output deep neural network was selected as the only surrogate model. This choice is central to the revised framework. The purpose is not to determine which machine-learning family performs best, because that benchmarking question was already addressed in earlier work [29]. The DNN is instead used as a fixed nonlinear response surface that can evaluate a large number of candidate designs at negligible marginal cost after training. The input vector contains two one-hot variables for base asphalt, four one-hot variables for SBS type, and four continuous variables for SBS dosage, shear temperature, shear rate, and shear time. The encoded input dimension is therefore ten. The output layer contains four neurons corresponding to penetration, softening point, ductility, and viscosity at 135 °C.

The 864 complete records were divided into training, validation, and test subsets using a fixed random seed of 42. The split proportions were 70%, 15%, and 15%, producing 604 training records, 130 validation records, and 130 test records. The balanced factorial structure of the database means that random splitting retains broad coverage of the material and processing combinations in each subset. All scaling statistics were calculated using the training subset only. The four continuous inputs were standardized, while one-hot indicators were left in binary form. The four output labels were also standardized during network training to prevent high-magnitude targets from dominating the loss.

For a continuous variable $x_{j}$, the standardized value $z_{j}$ was calculated using the training-set mean $\mu_{j}$ and standard deviation $\sigma_{j}$ as follows.

(1) $$z_{j}=\frac{x_{j}-\mu_{j}}{\sigma_{j}}$$

The same training-set parameters were then applied to validation, test, and optimization candidates. This procedure prevents information leakage from the test data and ensures that every NSGA-II candidate is transformed consistently before DNN inference.

#### 4.3.2. Network Architecture and Training

**Figure 15:**
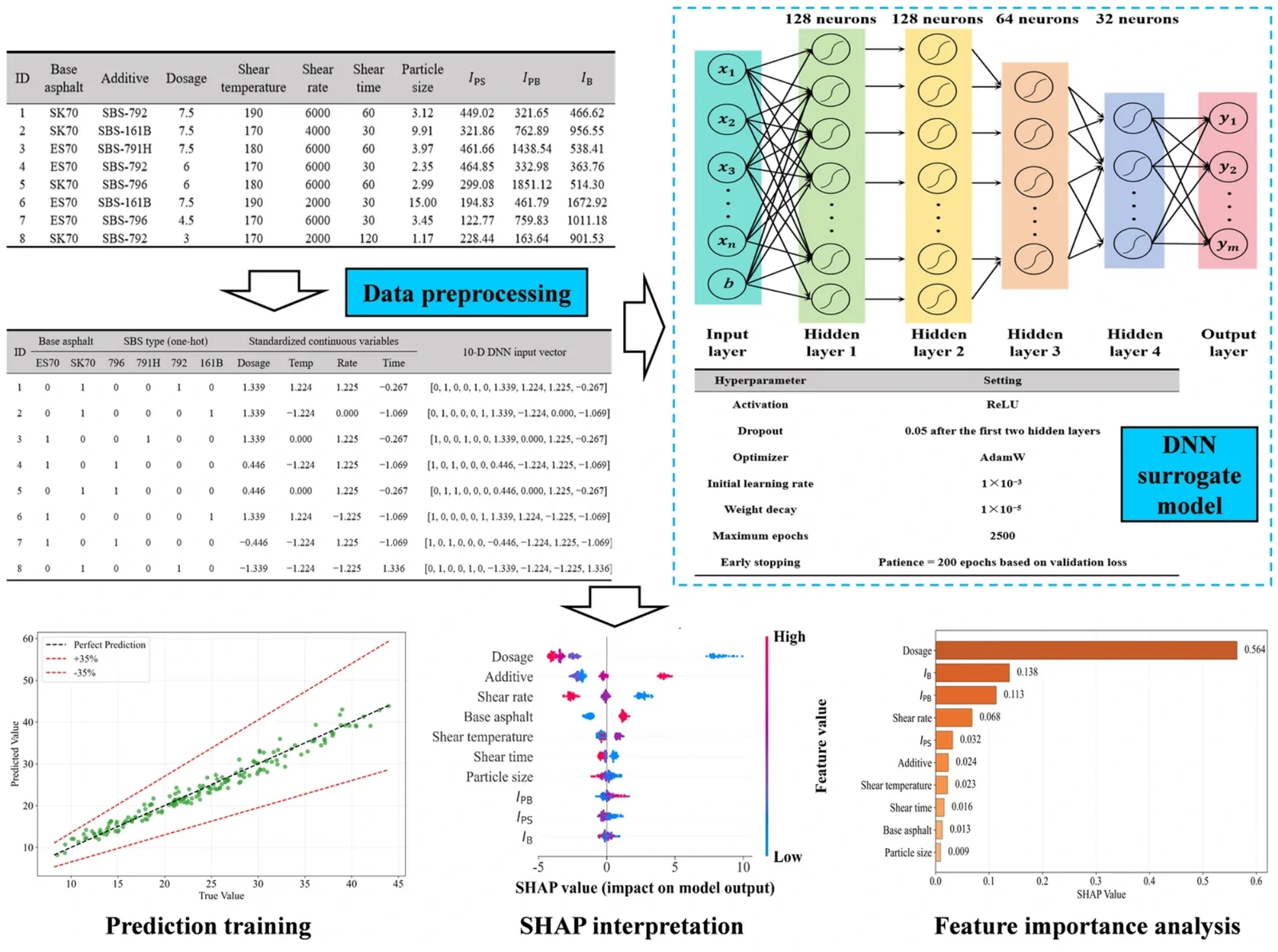
Framework and network architecture of the multi-output deep neural network surrogate.

The overall architecture, data preprocessing pipeline, training procedure and SHAP interpretability analysis for the multi-output DNN surrogate are visualized in Figure 15. The DNN consisted of an input layer followed by hidden layers with 128, 128, 64, and 32 neurons. Rectified linear unit activation was used after each hidden affine transformation, and a dropout rate of 0.05 was applied to the first two hidden layers. The final layer was linear and returned the four standardized target predictions simultaneously. Feedforward neural networks are universal function approximators under broad conditions [51], which makes them suitable for learning a smooth interpolation of the observed nonlinear response surface. In the present application, the multi-output structure also allows all four properties to share an internal representation of the same material and process variables instead of training four unrelated predictors. The DNN surrogate was implemented in Python 3.10.12. The network was built using TensorFlow 2.13.0 and Keras 2.13.1. Data preprocessing and scaling were performed with scikit-learn 1.2.0, NumPy 1.24.3, and pandas 2.0.3. All figures were generated with Matplotlib 3.7.2.

If $h^{\left(l\right)}$ denotes the hidden representation at layer l, the feedforward mapping can be written in compact form as follows.

(2) $$h^{\left(l\right)}=ReLU\left(W^{\left(l\right)}h^{\left(l-1\right)}+b^{\left(l\right)}\right),\;\hat{y}=W^{\left(L+1\right)}h^{\left(L\right)}+b^{\left(L+1\right)}$$

The network was trained with the mean squared error averaged over samples and the four standardized outputs. For n samples and q=4 targets, the loss was defined by Equation (3).

(3) $$L_{MSE}=\frac{1}{nq}\sum_{i=1}^{n}\sum_{k=1}^{q}{\left(y_{ik}-{\hat{y}}_{ik}\right)}^{2},\;q=4$$

Optimization used AdamW with an initial learning rate of $1\times 10^{-3}$ and weight decay of $1\times 10^{-5}$. AdamW decouples weight decay from the adaptive gradient step and is commonly used to improve regularization of deep networks [52]. Training was allowed to continue for a maximum of 2500 epochs. Validation loss was monitored after each epoch, and early stopping with a patience of 200 epochs was used to restore the parameter state associated with the minimum validation loss. This arrangement avoids selecting the final network from the test data.

The chosen architecture is intentionally moderate relative to the size of the database. The goal is a stable surrogate, not a highly over-parameterized representation. Dropout, weight decay, validation-based early stopping, and output standardization were used together to reduce overfitting risk. The complete network architecture and training configuration are summarized in Table 6. Deep-learning principles for supervised regression emphasize that model capacity should be judged by generalization to held-out data rather than by training loss alone [53]. The test subset was therefore used only after the architecture and stopping point had been fixed.

#### 4.3.3. Surrogate Evaluation Metrics

Surrogate accuracy was evaluated independently for each physical property on the 130-record test subset. Three metrics were used so that both relative explanatory power and absolute prediction error could be assessed. The coefficient of determination R^2^ measures how much of the observed variance is reproduced by the predictions. Root mean squared error gives greater weight to large deviations, while mean absolute error provides the average magnitude of the prediction residuals in the original engineering unit.

The coefficient of determination was calculated as Equation (4), where $y_{i}$ is the measured value, ${\hat{y}}_{i}$ is the DNN prediction, and y-bar is the sample mean.

(4) $$R^{2}=1-\frac{\sum_{i=1}^{n}{\left(y_{i}-{\hat{y}}_{i}\right)}^{2}}{\sum_{i=1}^{n}{\left(y_{i}-\bar{y}\right)}^{2}}$$

The two absolute-error metrics were calculated as follows.

(5) $$RMSE=\sqrt{\frac{1}{n}\sum_{i=1}^{n}{\left(y_{i}-{\hat{y}}_{i}\right)}^{2}}$$

(6) $$MAE=\frac{1}{n}\sum_{i=1}^{n}\left|y_{i}-{\hat{y}}_{i}\right|$$

The DNN was accepted as the optimization surrogate only after all four outputs showed stable test behavior and no property exhibited an error scale large enough to dominate the subsequent Pareto search. Because the dataset is a dense factorial interpolation domain, high held-out accuracy is expected to be more achievable than in a sparse field database. The limitation of this favorable data structure is considered explicitly in the Section 2.

### 4.4. NSGA-II Inverse Design

#### 4.4.1. Optimization Formulation

After training, all DNN weights and scaling parameters were frozen. The optimization stage therefore contains no further learning. NSGA-II generates candidate processing conditions, each candidate is encoded exactly as during DNN training, and the frozen network returns the four predicted properties. The algorithm then assigns dominance rank and crowding distance using those predictions. This strict separation between model training and optimization prevents the optimizer from altering the surrogate to favor particular regions of the design space.

The continuous decision vector was defined as x=[d, T, r, t], where d is SBS dosage, T is shear temperature, r is shear rate, and t is shear time. The bounds were 3.0% ≤ d ≤ 7.5%, 170 °C ≤ T ≤ 190 °C, 2000 rpm ≤ r ≤ 6000 rpm, and 30 min ≤ t ≤ 120 min. Softening point and ductility were maximized, while viscosity at 135 °C was minimized. NSGA-II is implemented as a minimization problem, so the objective vector was written as Equation (7).

(7) $$\underset{x}{min}\;F\left(x\right)=\left[\begin{array}{c} -{SP}_{DNN}\left(x\right)\\ -{Duc}_{DNN}\left(x\right)\\ {Vis}_{DNN}\left(x\right) \end{array}\right]$$

Penetration was predicted at every evaluation but was not included as a fourth objective. Its role was to provide an engineering consistency screen after the unconstrained Pareto search. The same approach was applied to viscosity. Although viscosity remained an explicit minimization objective, a practical reporting screen of viscosity no greater than 3.0 Pa·s was used when representative solutions were selected. Penetration between 45 and 70 in 0.1 mm was used as the corresponding consistency window. These values are not asserted here as universal specifications. They are used to prevent the engineering discussion from being dominated by extreme Pareto points that would be less representative of the central performance domain.

Base asphalt type and SBS type require separate treatment because they are categorical. Directly applying simulated binary crossover to integer category codes would create artificial intermediate materials. The search was therefore performed independently for each of the eight combinations formed by two base asphalts and four SBS types. Within a scenario, only the four continuous variables were evolved. The final non-dominated candidates from all eight searches were then pooled and subjected to a second dominance check. This scenario-wise strategy maintains the physical meaning of the categorical materials while still allowing a global comparison among their attainable Pareto fronts.

#### 4.4.2. Evolutionary Search Settings and Representative Solutions

NSGA-II used a population of 120 and 150 generations for each material scenario. Simulated binary crossover was applied with probability 0.90 and distribution index 15. Polynomial mutation used a per-variable probability of 0.25 and distribution index 20. The complete NSGA-II search configuration and reporting rules are summarized in Table 7. These settings preserve broad exploration while allowing local refinement as the population approaches the non-dominated region. The algorithmic logic follows the fast non-dominated sorting and elitist survival principles introduced for NSGA-II [37] and is consistent with established multi-objective evolutionary optimization practice [41,42]. A fixed random seed was used for reproducibility of the reported search. The NSGA-II search was implemented using pymoo 0.6.0 under Python 3.10.12. Convergence was evaluated separately for each material scenario using the hypervolume indicator. The three optimization objectives were first converted to a common minimization form and normalized using the same fixed bounds for all eight scenarios. Hypervolume was then calculated using a fixed reference point of (1.1, 1.1, 1.1) in the normalized objective space. The curves shown in Figure 3 therefore represent eight independent scenario-specific optimization runs rather than a pooled or aggregated convergence curve.

**Figure 16:**
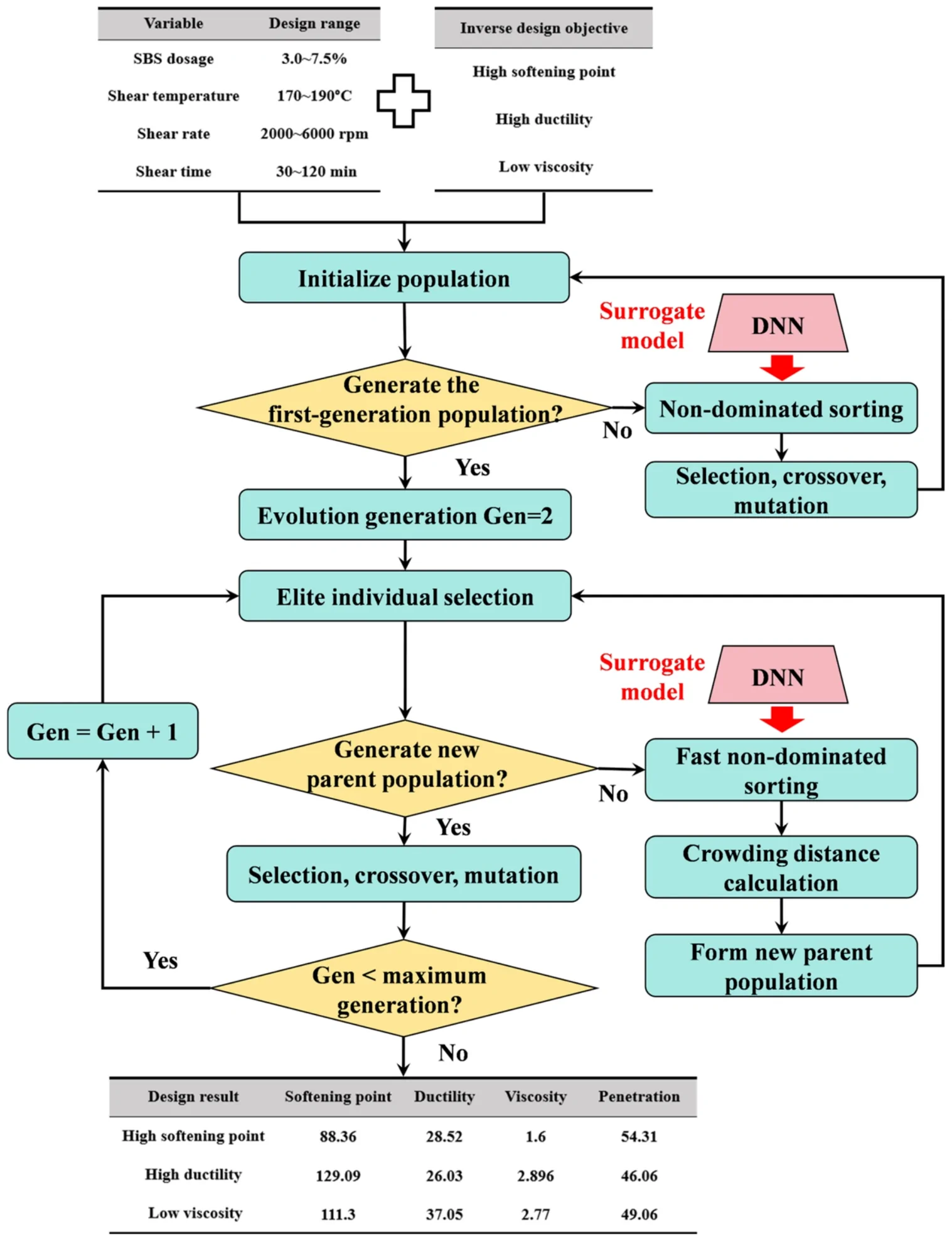
Flowchart for DNN surrogate-assisted NSGA-II inverse process design. The trained DNN model is frozen; multi-scenario evolutionary search, Pareto-solution pooling, screening and experimental validation are sequentially performed.

The full implementation logic of surrogate-assisted multi-objective inverse design is presented in Figure 16. No laboratory experiment is called during the evolutionary run. Once trained, the DNN evaluates an entire population in a small fraction of the time required to prepare even one physical binder. This is the operational benefit of the surrogate-assisted workflow. It does not eliminate experiments, because the final recommended continuous settings still require confirmation. Instead, it moves experiments from exhaustive search to targeted validation of a much smaller number of candidates.

A compromise solution was selected from the engineering-screened Pareto set by calculating distance to an ideal point after normalizing the three objectives over the retained front. Let $f_{im}$ be the value of objective m for solution i, and let the ideal and nadir values be determined from the retained Pareto set. The normalized Euclidean distance is given by Equation (8).

(8) $$d_{i}=\sqrt{\sum_{m=1}^{3}{\left(\frac{f_{im}-f_{m}^{ideal}}{f_{m}^{nadir}-f_{m}^{ideal}}\right)}^{2}}$$

The solution with the smallest $d_{i}$ was reported as the compromise design. Three additional points were selected to illustrate the extremes of high softening point, high ductility, and low viscosity. This representation is more informative than reporting one optimum because the Pareto set is intended to preserve different engineering preferences.

### 4.5. Comparison with Median Baseline Recipe and Experimental Validation

To quantify the improvement achieved by optimization, a median baseline recipe was defined as the median values of the four processing parameters from the training set: shear temperature of 180 °C, shear rate of 4000 rpm, shear time of 60 min, and SBS dosage of 4.5%. This recipe represents a typical empirical condition commonly used in practice. The performance of this recipe was predicted by the DNN surrogate and compared with that of the balanced optimal solution.

To confirm that the surrogate predictions generalize to the optimized regions of the design space, an additional sample was prepared using the recommended parameters from the balanced optimal solution. The sample was prepared following the same experimental protocols used for the dataset. The measured properties were compared with the predicted values. The relative errors between predicted and measured values were calculated to quantify the validation accuracy.

## Figures and Tables

**Table 1:** Test-set performance of the single multi-output DNN surrogate.

Target	R^2^	RMSE	MAE	P_95_(|e|)
Penetration, 0.1 mm	0.9811	0.196	0.150	0.382
Softening point, °C	0.9876	0.609	0.492	1.187
Ductility, cm	0.9939	0.270	0.213	0.533
Viscosity at 135 °C, Pa·s	0.9807	0.0357	0.0291	0.071

**Table 2:** Distribution of continuous decision variables among the 244 engineering-screened Pareto solutions.

Decision Variable	Median	25th Percentile	75th Percentile	Observed Screened Range
SBS dosage, %	3.468	3.111	5.488	3.000 to 6.346
Shear temperature, °C	181.137	179.234	185.035	173.256 to 190.000
Shear rate, rpm	4849.9	2046.5	5822.0	2000.0 to 5999.6
Shear time, min	54.71	31.46	112.59	30.00 to 120.00

**Table 3:** Representative designs selected from the screened Pareto set.

Design	Base Asphalt + SBS	Dosage %	T °C	Rate rpm	Time min	Pen. 0.1 mm	SP °C	Duc. cm	Visc. Pa·s
Balanced	ES70 + SBS-161B	3.242	179.1	4796	53.2	54.31	88.36	28.52	1.600
High SP	SK70 + SBS-161B	5.829	178.6	4555	113.4	46.06	129.09	26.03	2.896
High ductility	ES70 + SBS-796	5.912	178.8	5861	69.3	49.06	111.30	37.05	2.770
Low viscosity	SK70 + SBS-791H	3.000	190.0	2000	30.0	60.55	55.06	12.29	0.474

**Table 4:** Comparison of the representative optimized solutions in this study with reported SBS-modified asphalt performance in the literature. The ductility measurements were conducted at 5 °C with an extension rate of 5 cm/min.

Optimized Solutions	Softening Point (°C)	Ductility (5 °C, 5 cm/min) (cm)
Wang et al., 2020 [43]	89.3	39.2
Qian et al., 2021 [44]	79.0	34.0
Niu et al., 2023 [45]	67.0	30.1
Yi et al., 2025 [46]	80.3	39.2
Balanced	88.36	28.52
High softening point	129.09	26.03
High ductility	111.30	37.05
Low viscosity	55.06	12.29

**Table 5:** Data domain, optimization role, and statistical ranges in the supplied SBS-modified asphalt database.

Variable	Role in This Study	Levels or Range	Mean ± SD Where Applicable
Base asphalt	Discrete design scenario	ES70; SK70	—
SBS type	Discrete design scenario	SBS-796; SBS-791H; SBS-792; SBS-161B	—
SBS dosage, %	Continuous decision variable	3.0~7.5	5.25 ± 1.68
Shear temperature, °C	Continuous decision variable	170~190	180.0 ± 8.17
Shear rate, rpm	Continuous decision variable	2000~6000	4000 ± 1633
Shear time, min	Continuous decision variable	30~120	70.0 ± 37.42
Particle size, μm	Descriptive microstructure	0.513~17.048	4.808 ± 3.167
$I_{PS}$	Descriptive microstructure	46.399~897.398	256.758 ± 156.745
$I_{PB}$	Descriptive microstructure	80.904~2281.815	641.256 ± 422.796
$I_{B}$	Descriptive microstructure	200.324~5018.775	1215.291 ± 792.610
Penetration, 0.1 mm	DNN target label	37.647~75.468	50.400 ± 7.275
Softening point, °C	DNN target label	45.667~143.044	89.831 ± 21.565
Ductility, cm	DNN target label	7.086~47.547	23.543 ± 8.223
Viscosity at 135 °C, Pa·s	DNN target label	0.463~5.574	2.429 ± 1.185

**Table 6:** Configuration of the single multi-output DNN surrogate.

Item	Setting
Input descriptors	2 base-asphalt in one-hot format, 4 SBS-type in one-hot format, dosage, temperature, rate, time
Input dimension	10
Outputs	Penetration; softening point; ductility; viscosity at 135 °C
Hidden layers	128, 128, 64, 32 neurons
Activation	ReLU
Dropout	0.05 after the first two hidden layers
Optimizer	AdamW
Initial learning rate	$1\times 10^{-3}$
Weight decay	$1\times 10^{-5}$
Maximum epochs	2500
Early stopping	Patience = 200 epochs based on validation loss
Data split	70% training, 15% validation; 15% test; random seed = 42

**Table 7:** NSGA-II search configuration and reporting rules.

Component	Setting
Material scenarios	8 independent combinations: 2 base asphalts × 4 SBS types
Decision variables	SBS dosage, shear temperature, shear rate, shear time
Population size	120 per scenario
Generations	150 per scenario
Crossover	Simulated binary crossover; probability 0.90; distribution index 15
Mutation	Polynomial mutation; probability 0.25 per variable; distribution index 20
Objectives	Maximize softening point; maximize ductility; minimize viscosity at 135 °C
Additional predicted property	Penetration
Engineering reporting screen	45 ≤ penetration ≤ 70 in 0.1 mm; viscosity ≤ 3.0 Pa·s
Compromise selection	Minimum normalized Euclidean distance to ideal point

## Data Availability

The data that support the findings of this study are available from the authors upon reasonable request.
